# Aggressive B cell lymphomas retain ATR-dependent determinants of T cell exclusion from the germinal center dark zone

**DOI:** 10.1172/JCI187371

**Published:** 2025-07-17

**Authors:** Valeria Cancila, Giorgio Bertolazzi, Allison S.Y. Chan, Giovanni Medico, Giulia Bastianello, Gaia Morello, Daniel Paysan, Clemence Lai, Liang Hong, Girija Shenoy, Patrick W. Jaynes, Giovanna Schiavoni, Fabrizio Mattei, Silvia Piconese, Maria V. Revuelta, Francesco Noto, Luca Businaro, Adele De Ninno, Ilenia Cammarata, Fabio Pagni, Saradha Venkatachalapathy, Sabina Sangaletti, Arianna Di Napoli, Giada Cicio, Davide Vacca, Silvia Lonardi, Luisa Lorenzi, Andrés J.M. Ferreri, Beatrice Belmonte, Min Liu, Manikandan Lakshmanan, Michelle S.N. Ong, Biyan Zhang, Tingyi See, Kong-Peng Lam, Gabriele Varano, Mario P. Colombo, Silvio Bicciato, Giorgio Inghirami, Leandro Cerchietti, Maurilio Ponzoni, Roberta Zappasodi, Evelyn Metzger, Joseph Beechem, Fabio Facchetti, Marco Foiani, Stefano Casola, Anand D. Jeyasekharan, Claudio Tripodo

**Affiliations:** 1 Tumor Immunology Unit, Department of Health Sciences, University of Palermo, Palermo, Italy.; 2 Department of Medicine and Surgery, Kore University of Enna, Enna, Italy.; 3 Cancer Science Institute of Singapore, National University of Singapore, Singapore.; 4 Division of Hematopathology, Department of Pathology and Laboratory Medicine, Weill Cornell Medicine and NewYork-Presbyterian Hospital, New York, New York, USA.; 5 Genome Integrity Lab, IFOM ETS–The Italian Association for Cancer Research (AIRC) Institute of Molecular Oncology, Milan, Italy.; 6 Laboratory for Nanoscale Biology, Paul Scherrer Institute, Villigen, Switzerland.; 7 Department of Health Sciences and Technology, ETH Zürich, Zürich, Switzerland.; 8 Department of Oncology and Molecular Medicine, Istituto Superiore di Sanità, Rome, Italy.; 9 Department of Internal Clinical Sciences, Anesthesiology and Cardiovascular Sciences, Sapienza University of Rome, Rome, Italy.; 10 Istituto di Ricovero e Cura a Carattere Scientifico (IRCCS) Fondazione Santa Lucia, Unità di Neuroimmunologia, Rome, Italy.; 11 Laboratory affiliated to Istituto Pasteur Italia–Fondazione Cenci Bolognetti, Rome, Italy.; 12 Division of Hematology and Oncology, Medicine Department, Weill Cornell Medicine and NewYork-Presbyterian Hospital, New York, New York, USA.; 13 Institute for Photonics and Nanotechnologies, Italian National Research Council, Rome, Italy.; 14 Department of Translational and Precision Medicine, Sapienza University of Rome, Neuroimmunology Unit, IRCCS Fondazione Santa Lucia, Rome, Italy.; 15 Department of Medicine and Surgery, Pathology, IRCCS Fondazione San Gerardo dei Tintori, University of Milano–Bicocca, Milan, Italy.; 16 Molecular Immunology Unit, Department of Experimental Oncology, Fondazione IRCCS Istituto Nazionale Tumori, Milan, Italy.; 17 Pathology Unit, Department of Clinical and Molecular Medicine, Sant’Andrea University Hospital, Sapienza University of Rome, Rome, Italy.; 18 Advanced Pathology Laboratory, IFOM ETS–The AIRC Institute of Molecular Oncology, Milan, Italy.; 19 Pathology Unit, ASST Spedali Civili di Brescia, Department of Molecular and Translational Medicine, University of Brescia, Brescia, Italy.; 20 Lymphoma Unit, IRCCS San Raffaele Scientific Institute, Milan, Italy.; 21 Vita-Salute San Raffaele University, Milan, Italy.; 22 Department of Radiation Oncology, Chongqing University Cancer Hospital, Chongqing, China.; 23 Department of Immunology, Tianjin Medical University Cancer Institute and Hospital, Tianjin, China.; 24 Institute of Molecular and Cell Biology, Agency for Science, Technology and Research (A*STAR), Singapore.; 25 Singapore Immunology Network, A*STAR, Singapore.; 26 Department of Microbiology and Immunology, Immunology Translational Research Programme, Yong Loo Lin School of Medicine, National University of Singapore, Singapore.; 27 School of Biological Sciences, Nanyang Technological University, Singapore.; 28 Genetics of B Cells and Lymphoma Unit, IFOM ETS–The AIRC Institute of Molecular Oncology, Milan, Italy.; 29 Department of Molecular Medicine, University of Padova, Padova, Italy.; 30 Pathology Unit, IRCCS San Raffaele Scientific Institute, Milan, Italy.; 31 Department of Medicine, Weill Cornell Medicine, New York, New York, USA.; 32 Bruker Spatial Biology, Seattle, Washington, USA.; 33 Department of Medical Biotechnology and Translational Medicine, University of Milan, Milan, Italy.; 34 Department of Haematology-Oncology, National University Health System, Singapore.; 35 NUS Centre for Cancer Research, Yong Loo Lin School of Medicine, and; 36 Department of Medicine, Yong Loo Lin School of Medicine, National University of Singapore, Singapore.; 37 Department of Oncology and Hemato-Oncology, University of Milan, Milan, Italy.

**Keywords:** Cell biology, Immunology, Oncology, Lymphomas, Molecular pathology, T cells

## Abstract

The germinal center (GC) dark zone (DZ) and light zone represent distinct anatomical regions in lymphoid tissue where B cell proliferation, immunoglobulin diversification, and selection are coordinated. Diffuse large B cell lymphomas (DLBCLs) with DZ-like gene expression profiles exhibit poor outcomes, though the reasons are unclear and are not directly related to proliferation. Physiological DZs exhibit an exclusion of T cells, prompting exploration of whether T cell paucity contributes to DZ-like DLBCL. We used spatial transcriptomic approaches to achieve higher resolution of T cell spatial heterogeneity in the GC and to derive potential pathways that underlie T cell exclusion. We showed that T cell exclusion from the DZ was linked to DNA damage response (DDR) and chromatin compaction molecular features characterizing the spatial DZ signature, and that these programs were independent of activation-induced cytidine deaminase (AID) activity. As ATR is a key regulator of DDR, we tested its role in the T cell inhibitory DZ transcriptional imprint. ATR inhibition reversed not only the DZ transcriptional signature, but also DZ T cell exclusion in DZ-like DLBCL in vitro microfluidic models and in in vivo samples of murine lymphoid tissue. These findings highlight that ATR activity underpins a physiological scenario of immune silencing. ATR inhibition may reverse the immune-silent state and enhance T cell–based immunotherapy in aggressive lymphomas with GC DZ–like characteristics.

## Abstract 2

Inhibition of ATR reverses the dark zone signature linked to T-cell exclusion in aggressive B-cell lymphomas.

## Graphical abstract

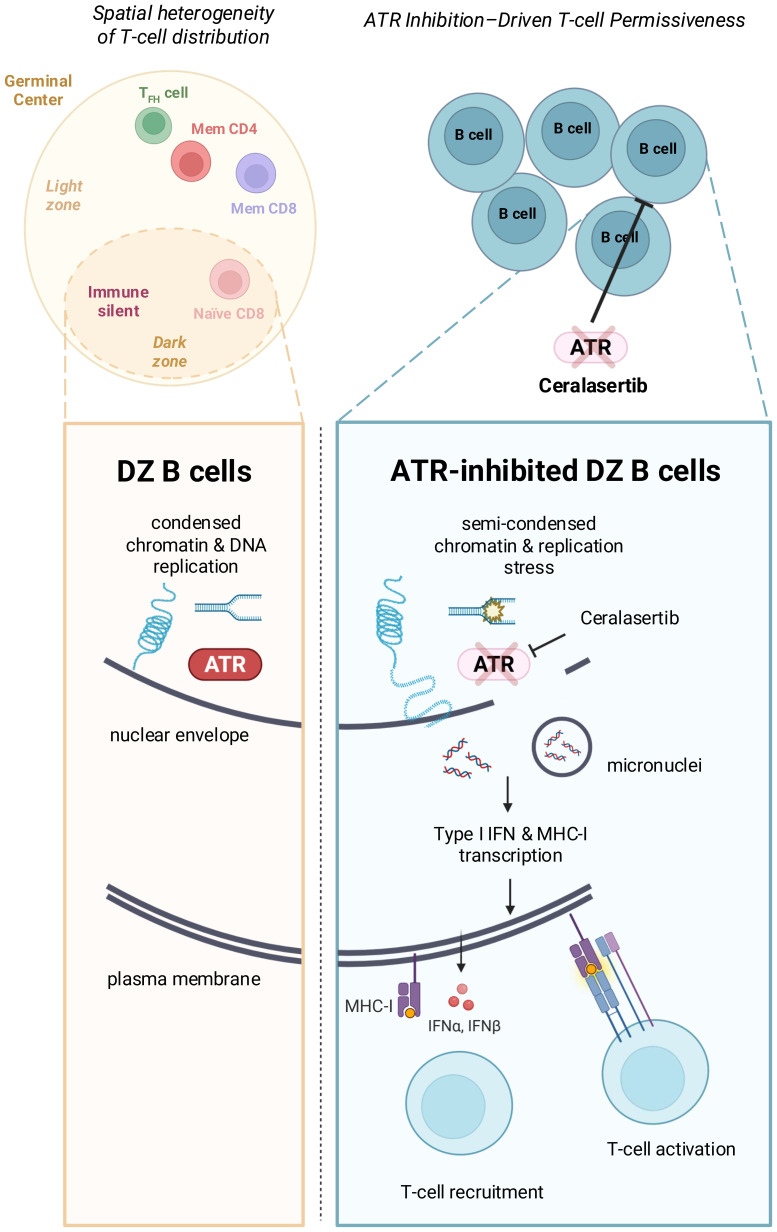

## Introduction

Tumors employ diverse strategies to evade the immune system, ranging from altering their intrinsic properties to manipulating their microenvironment (1). Evasion mechanisms include tumor cell expression of inhibitory immune checkpoint molecules (2), recruitment of immunosuppressive cell subsets (3, 4), and modification of the extracellular matrix to impede immune cell function (5, 6). T cells contribute to antitumor immunity and are also central to multiple immunotherapy approaches, including checkpoint blockade and adoptive cell therapies (1). Understanding the mechanisms that govern T cell exclusion within the tumor microenvironment is essential for improving treatment response and immunotherapeutic efficacy.

In epithelial cancers, T cell exclusion has been linked to stromal remodeling and activation of cell-intrinsic pathways such as WNT/β-catenin, TGF-β, and PI3K signaling (7–11). In contrast, the mechanisms underlying T cell exclusion in hematological malignancies like diffuse large B cell lymphoma (DLBCL) remain poorly defined. Unlike epithelial tumors, lymphomas lack structured tumor-stroma boundaries, posing challenges in delineating spatial immune barriers. While several immune escape mechanisms in lymphoma have been identified, including loss of MHC molecules and constitutive PD-L1 expression (12, 13), the complete range of strategies these lymphomas use to evade T cell–mediated immunity is still unclear. Lymphomas with a double-hit gene signature (DHITsig) and poor prognosis display fewer T cells (14, 15), but the mechanisms of this are also not fully defined. Similarly, dark zone–like lymphomas resemble the dark zone of the germinal center (GC); thus we turned to GCs in lymphoid organs as a model system to explore the basis of T cell exclusion in a homogeneous cellular context (16–18). Although not physically separated, the GC is functionally organized into 2 distinct regions: the dark zone (DZ), where B cells proliferate and mutate their antibody variable genes, and the light zone (LZ), where B cells engage other immune cells, including T follicular helper cells, to refine antibody affinity (19, 20). This natural segregation of cellular activities within the GC provides an ideal setting to study factors influencing T cell distribution.

Using quantitative immunohistochemistry (IHC), digital spatial profiling (DSP), and single-cell spatial transcriptomics, we examined the in situ microenvironment of the GC LZ and DZ areas, detailing their relationship with T cell localization and phenotype. We identified the LZ-DZ interface as a barrier-less constraint limiting homogeneous intra-GC T cell distribution. T cell exclusion was recapitulated in DLBCLs expressing a GC DZ gene signature, suggesting a link between GC biology and tumor immune evasion. Spatial transcriptomics analysis revealed preferential activation of DNA damage response (DDR) pathways and suppression of inflammatory signaling in the DZ, correlating with increased chromatin compaction. We identify the ataxia telangiectasia and Rad3 related (ATR) kinase as a central regulator of these pathways, sustaining the DZ transcriptional program and promoting T cell exclusion. These findings position ATR as a central player in spatial regulation of the GC reaction and point to potential mechanisms that may be co-opted by a subset of DLBCLs to facilitate immune evasion.

## Results

### Spatial profiling reveals distinct transcriptional programs in GC DZ and LZ regions.

**Figure 1:**
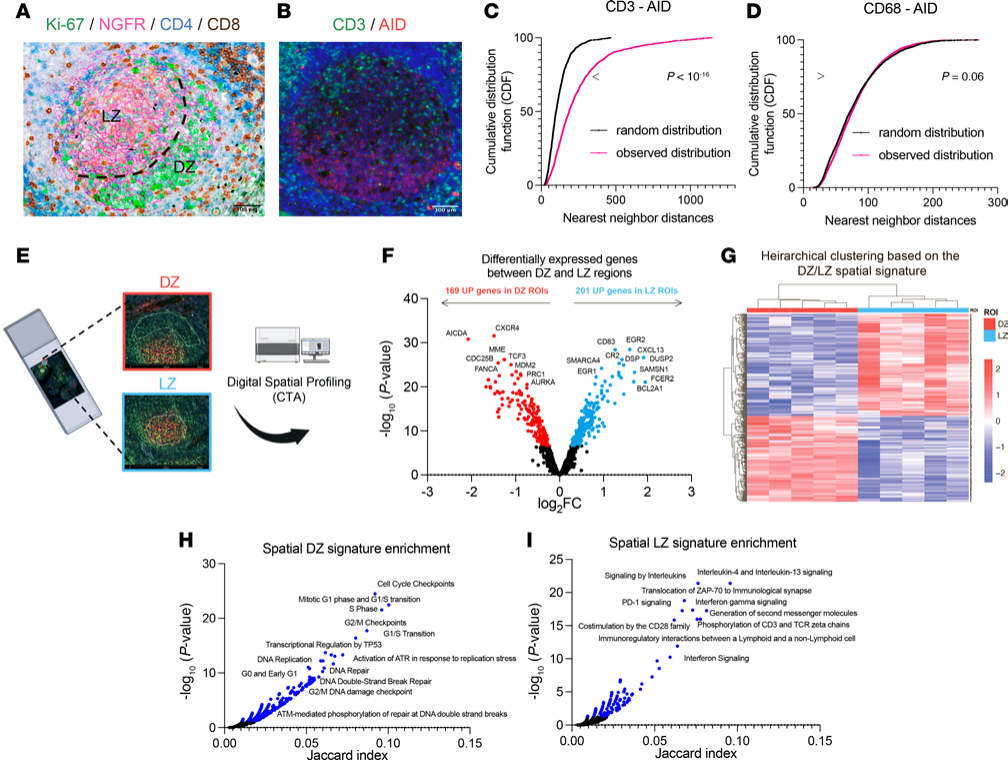
Spatial profiling uncovers unique transcriptional programs in the dark and light zones of GCs. (**A**) Representative immunohistochemistry/immunofluorescence (IHC/IF) micrographs showing Ki-67 (green signal), NGFR (pink signal), CD4 (blue signal), and CD8 (brown signal) expression. Ki-67 highlights proliferative DZ regions; NGFR marks the LZ. Original magnification, ×200. Scale bar: 100 μm. (**B**) Representative IF images of CD3^+^ (green signal) and AID^+^ (red signal) cells within GCs. Original magnification, ×200. Scale bar: 100 μm. (**C**) Cumulative distribution functions (CDFs) of CD3^+^–AID^+^ nearest-neighbor distances in observed samples (pink curve) versus randomized controls (black curve). Statistical analysis: Wilcoxon’s test. (**D**) CDFs of CD68^+^–AID^+^ nearest-neighbor distances in observed versus randomized samples. Statistical analysis: Wilcoxon’s test. (**E**) DSP analysis of ROIs from DZ (*n* = 5) and LZ (*n* = 5) regions defined by CD20 and NGFR expression. (**F**) Volcano plot showing differentially expressed genes (adjusted *P* < 0.05) between DZ and LZ regions. (**G**) Heatmap of differentially expressed genes with unsupervised hierarchical clustering across ROIs. (**H**) Pathway enrichment of 169 DZ-upregulated genes using the Reactome Pathway database. (**I**) Pathway enrichment of 201 LZ-upregulated genes using Reactome.

Reactive GCs display spatial compartmentalization, with a proliferative, Ki-67–dense DZ and a follicular dendritic cell–rich (FDC-rich) LZ, bordered by T cell–dense extrafollicular regions (Figure 1A). Spatial analysis highlighted significant exclusion of CD3^+^ T cells from the activation-induced cytidine deaminase–positive (AID-marked) DZ, evidenced by an increased CD3-to-AID distance compared with a random spatial distribution (Figure 1, B and C, and Supplemental Figure 1A; supplemental material available online with this article; https://doi.org/10.1172/JCI187371DS1). In contrast, CD68^+^ macrophages were uniformly distributed across both zones (Figure 1D and Supplemental Figure 1, B and C), serving as a control that supports the specificity of T cell exclusion from the DZ.

To investigate the molecular programs underlying T cell exclusion from the DZ, we performed spatial transcriptional profiling of reactive GCs. DZ and LZ regions were defined using the B cell marker CD20 and the LZ marker CD271/NGFR (Figure 1E). Digital spatial profiling was chosen for its ability to capture spatially resolved gene expression while preserving tissue architecture. Using the GeoMx Cancer Transcriptome Atlas, we analyzed 1,824 immune- and tumor-related genes in matched GC DZ and LZ regions from human tonsils (*n =* 10 pairs) (21).

This analysis identified a robust DZ/LZ transcriptional signature composed of 370 differentially expressed genes (adjusted *P* < 0.05), with 169 upregulated in the DZ and 201 in the LZ (Figure 1, F and G, and Supplemental Table 1). Pathway enrichment revealed functionally distinct profiles: DZ regions were enriched for DDR, cell cycle progression, and DNA replication stress, while LZ regions were enriched for immune signaling pathways (Figure 1, H and I, and Supplemental Table 2). Given their spatial origin, our DZ and LZ signatures encompass transcripts from multiple cell types. To validate that these signatures adequately represent the biology of DZ and LZ B cells, we applied them to a whole-transcriptome single-cell RNA sequencing (scRNA-Seq) dataset of GC B cells (Gene Expression Omnibus GSE139891) (Supplemental Figure 1D) (17). The signatures effectively distinguished DZ from LZ B cell populations, confirming that they also capture B cell transcriptional states. While this provides orthogonal validation, the primary strength of our approach lies in its spatial resolution, enabling analysis across the full tissue context — including non–B cell contributions.

As an additional validation, we performed an independent whole-transcriptome atlas (WTA) DSP and compared DZ and LZ regions. We used 2 gene lists to classify GC B cells into DZ and LZ types: the spatial DZ/LZ signature and the differentially expressed genes identified through the WTA DSP. The spatial DZ/LZ signature showed greater predictive power than the WTA differentially expressed genes, confirming its strong discriminatory capacity (Supplemental Figure 1E).

### Spatial heterogeneity of T cell distribution within the GC.

**Figure 2:**
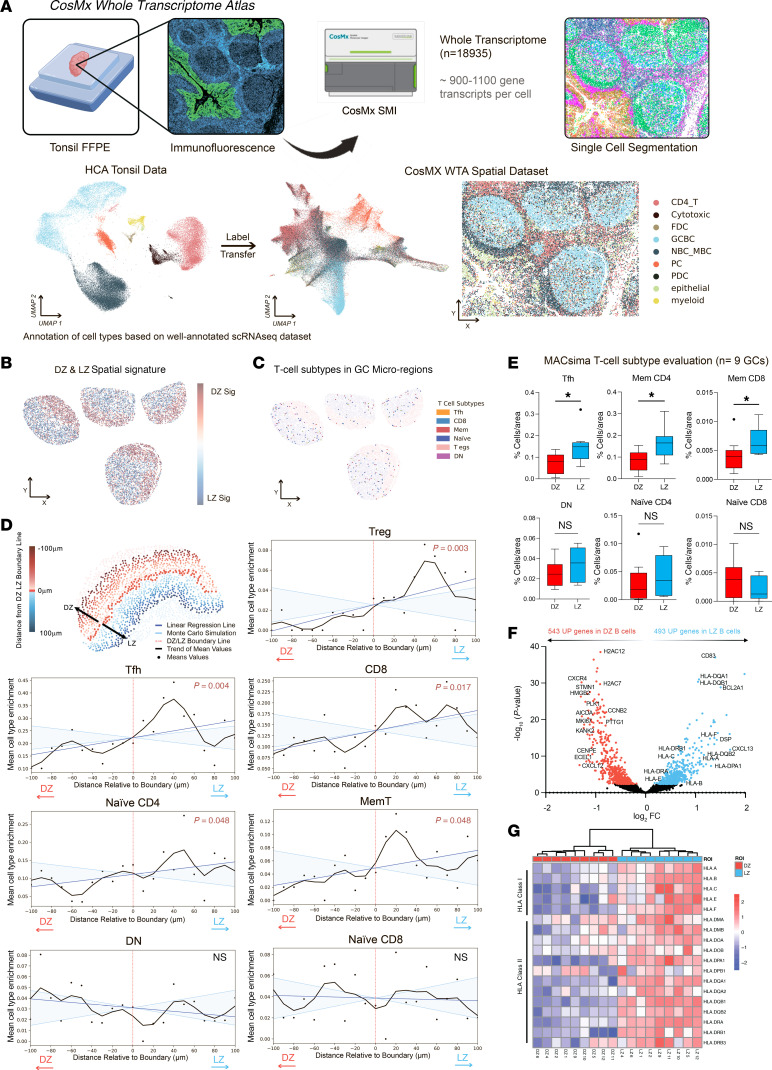
T cell distribution in the GC varies by subset and function. (**A**) Schematic overview of the CosMx SMI whole-transcriptome (WTX) workflow. FFPE tonsil tissues were processed, followed by IF imaging and single-cell segmentation. Spatial transcriptomics was performed for 18,935 RNA targets, detecting approximately 900–1,100 transcripts per cell. Data were visualized by uniform manifold approximation and projection (UMAP), and cell type identities were assigned via label transfer from the HCA tonsil reference dataset. (**B**) Spatial enrichment maps of DZ and LZ transcriptional signatures across 4 representative GCs. (**C**) Spatial distribution of T cell subtypes in GC microregions, highlighting immune cells including Tfh, CD8^+^, memory, naive CD8^+^, Treg, T helper, γδ T, T follicular regulatory, and DN cells. (**D**) Quantification of T cell distribution relative to the DZ-LZ boundary. Cells were analyzed within a –100 μm (DZ) to 100 μm (LZ) range, binned into 10-μm increments. Subtypes analyzed include Treg, Tfh, CD8^+^, naive CD4^+^, memory T, DN, and naive CD8^+^, with spatial trends depicted in a line graph. (**E**) Quantification of T cell subtypes including Tfh, memory CD4^+^, memory CD8^+^, DN, naive CD4^+^, and naive CD8^+^ cells based on MACSima hyperplex analyses to evaluate their differential distribution between DZ and LZ (*n* = 9 GCs). Statistical analysis was assessed using a 2-tailed unpaired Mann-Whitney test. Values are shown as mean ± SEM; *P < 0.05. (**F**) Volcano plot of differentially expressed genes between DZ and LZ B cells, highlighting upregulated genes in each region (adjusted *P* value < 0.05). (**G**) Heatmap of HLA class I and II gene expression in DZ versus LZ regions, clustered hierarchically by expression pattern.

To investigate the spatial organization of T cell subsets in lymphoid tissue, we applied a single-cell-resolved spatial whole-transcriptome (WTX) approach using the CosMx Spatial Molecular Imager (SMI) platform across 9 relative normal human tonsillar GCs. Previous iterations of CosMx SMI enabled profiling of up to 6,000 RNA targets; the latest version allows detection of over 18,000 transcripts, enabling comprehensive spatial transcriptomics at both single-cell and subcellular resolution. Cell type annotation was performed via label transfer from a well-annotated scRNA-Seq reference dataset (HCATonsilData) using MaxFuse, a cross-modality integration algorithm. This strategy enabled robust identification of transcriptionally defined immune populations — including T regulatory (Treg), T follicular helper (Tfh), memory, CD8^+^ effector, naive CD8^+^, and CD4/CD8-double-negative (DN) T cells — within the architectural context of the GC (Figure 2A) (22).

DZ and LZ regions were independently segmented based on spatial transcriptomic signatures. We first confirmed that the DSP-derived DZ and LZ signatures could accurately define corresponding regions within the CosMx datasets, providing orthogonal spatial validation across platforms (Figure 2B and Supplemental Figure 2A). With these defined compartments, we next investigated the distribution of T cell subsets within each region, enabling precise mapping of immune cell populations relative to the DZ-LZ boundary (Figure 2C). Most active T cell subsets — Tregs, Tfh cells, CD8^+^ T cells, and memory T cells — were enriched in the LZ. In contrast, naive CD8^+^ and DN T cells showed no compartmental preference (Figure 2D). Naive CD8^+^ T cells were defined by high expression of CD8 and CCR7, and low expression of activation- and memory-associated genes, including HLA-DRB1, GZMA, LAG3, IFNG, and CD99; all non-naive CD8^+^ cells were classified as CD8^+^ T cells.

The spatial trends were corroborated at the protein level using hyperplexed immunofluorescence (IF) with the MACSima platform, which demonstrated significant LZ enrichment for Tfh and memory T cell subsets (CD4^+^ and CD8^+^), while DN and naive CD4^+^CD8^+^ T cells showed no significant association with either compartment (Figure 2E and Supplemental Figure 2, B and C). The lack of LZ enrichment for naive CD8^+^ and DN T cells — populations not expected to be actively engaged in MHC-dependent interactions — was consistent with the reduced expression of MHC molecules in DZ B cells compared with those in the LZ (Figure 2, F and G, and Supplemental Table 3).

Since DN T cells include a substantial fraction of γδ T cells, we further examined their localization within GCs. IHC for γδTCR (T cell receptors), a marker of γδ T cells, revealed rare γδ T cells, which were preferentially enriched in the DZ (Supplemental Figure 2, D and E) (23).

To further assess the spatial positioning of T cells relative to B cell subtypes, we quantified the proximity of CD4^+^ and CD8^+^ T cells to PLK1^+^ DZ B cells and EGR1^+^ LZ B cells — representative markers derived from our spatial transcriptional signatures (Supplemental Figure 2, F–I). Both T cell types were located closer to LZ B cells, consistent with their LZ enrichment. However, CD8^+^ T cells exhibited markedly shorter distances to DZ B cells than CD4^+^ T cells. This proximity correlated with increased expression of IFN-γ among CD8^+^ T cells, with IFN-γ^+^ cells preferentially localized at the DZ-LZ interface (Supplemental Figure 2, J–L), suggesting that effector CD8^+^ T cells accumulate at GC boundaries where they may be poised for functional engagement.

Together, these findings reveal compartment-specific localization of T cell subsets, with functional CD8^+^ T cells concentrating at the GC interface and naive or DN T cells distributing independently of GC zonation.

### The GC DZ spatial signature negatively correlates with T cell gene programs and clinical outcome in DLBCL.

**Figure 3:**
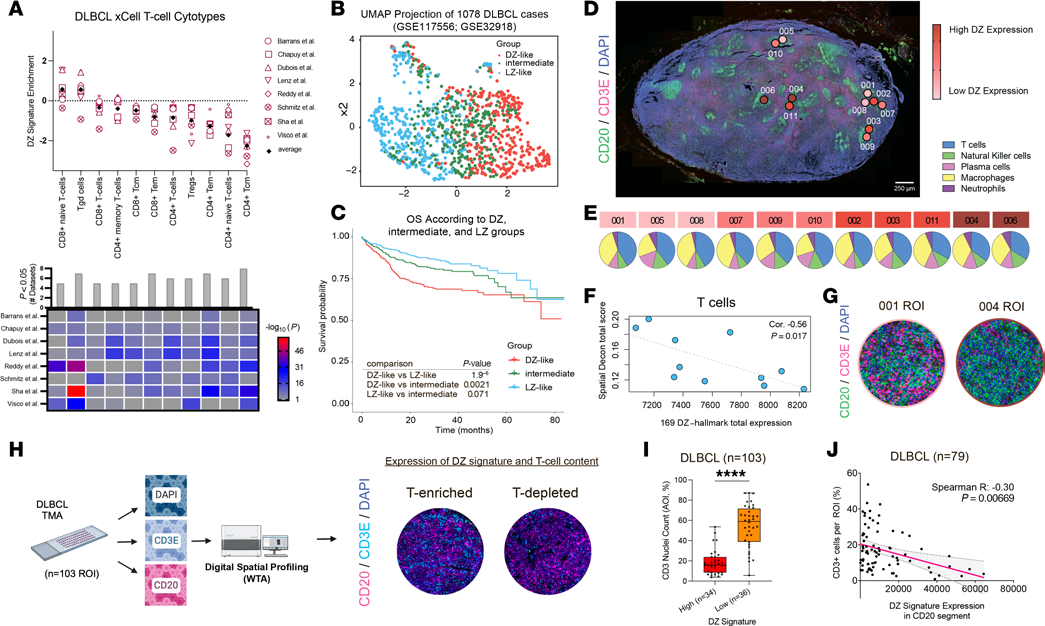
The GC DZ spatial signature in aggressive B cell lymphomas is associated with reduced T cell infiltration. (**A**) DZ enrichment scores correlating DZ gene expression and xCell T cell cytotype scores calculated in 8 DLBCL datasets. Positive DZ enrichment values indicate a positive association between the DZ spatial signature and the xCell cytotype scores, while negative values indicate a negative association. Statistical significance is shown with Wilcoxon’s adjusted *P* values. (**B**) UMAP projection of 1,078 harmonized DLBCL cases classified based on the DZ/LZ spatial signature; DZ-like cases (red), LZ-like cases (light blue), intermediate cases (green). (**C**) Kaplan-Meier survival plot showing overall survival (OS) of DZ-like, LZ-like, and intermediate groups from the harmonized dataset (1,078 cases). (**D**) DSP images of 11 ROIs selected within CD20 (green signal) and CD3E (red signal) infiltrates of a lymph node with DLBCL. Total expression of the DZ signature is shown from low (pink) to high (red). Original magnification, ×50. Scale bar: 250 μm. (**E**) Pie charts showing SpatialDecon cytotype scores across 11 ROIs, ranked by DZ signature expression. (**F**) Scatterplot with correlation line of DZ spatial signature expression and SpatialDecon T cell score across 11 ROIs (Kendall’s correlation, *P* < 0.05). (**G**) DSP images of lowest DZ signature expression ROI (001) and highest DZ signature expression ROI (004). (**H**) Schematic of DLBCL tissue microarray (TMA) consisting of 103 patient samples, analyzed using DSP WTA with DAPI, CD20 (red signal​), and CD3E (cyan signal) to observe T cell content and DZ signature expression. (**I**) Box plot comparing CD3 AOI nuclear count percentages between high and low DZ signature groups. Statistical analysis was performed using a 2-tailed unpaired Mann-Whitney test. Values are shown as mean ± SEM; ****P < 0.0001. (**J**) Scatterplot with correlation line of DZ spatial signature expression within the CD20^+^ segment and the percentage of CD3^+^ cells per ROI across 79 DLBCL samples. Statistical significance was assessed using Spearman’s correlation coefficient (*R*) and *P* value.

DZ-associated gene signatures, including the double-hit signature (DHITsig) and the molecular high-grade (MHG) signature, are linked to high-grade B cell lymphomas with poor prognosis and reduced T cell abundance (14, 15). DHITsig was originally identified in high-grade B cell lymphoma with MYC and BCL2 rearrangements (HGBCL-DH-BCL2). We have previously shown that a spatial GC DZ signature clusters aggressive lymphoma types (MHG and DHIT) and correlates with poor prognosis (18). To investigate whether lymphomas exhibiting high DZ spatial signature expression display similar T cell depletion patterns, we analyzed transcriptomic data from 3,610 DLBCL cases across 8 independent cohorts (GSE32918, GSE98588, GSE87371, GSE10846, Reddy, Schmitz, GSE117556, GSE31312) (24–31). Using xCell transcriptional deconvolution (32), we found that DZ-like cases had reduced frequencies of most T cell populations, except for CD8^+^ and γδ T cells, recapitulating the patterns observed in reactive GCs (Figure 3A).

Next, we assessed the prognostic significance of the DZ and LZ spatial signatures by analyzing transcriptomic data from 1,078 aggressive B cell lymphomas, harmonized from 2 well-annotated RNA-Seq datasets (GSE117556 and GSE32918). Based on the expression of the DZ/LZ spatial signature, we classified the samples into DZ-like, LZ-like, and intermediate groups. Uniform manifold approximation and projection (UMAP) of these groups confirmed distinct expression patterns (Figure 3B). DZ-like cases were associated with significantly shorter overall survival (Figure 3C and Supplemental Figure 3A), a trend that remained consistent across GC B cell (GCB) and activated B cell (ABC) subtypes (Supplemental Figure 3, B–E). Analysis of 35 DHL cases revealed that those with a high DZ signature exhibited the lowest expression of the T cell signature, further supporting that the GC DZ signature captures a DZ-like biology in aggressive B cell lymphomas involving attenuated T cell infiltration (Supplemental Figure 3F).

To determine whether the inverse correlation between DZ spatial signature expression and T cell infiltration holds true at the level of intratumoral heterogeneity, we performed DSP on 11 regions of interest from a single DLBCL lymph node biopsy (Figure 3D and Supplemental Table 4). Immune cell composition analysis revealed a strong inverse correlation between DZ signature expression and T cell abundance (Figure 3, E and F), with no significant associations detected for other immune cell populations (Supplemental Figure 3G). Visualization of CD3 staining showed reduced T cell density in regions with high DZ signature expression, supporting the inverse correlation (Figure 3G).

To assess whether this spatial relationship holds across a larger cohort, we analyzed an independent cohort of 103 DLBCL tissue microarray samples profiled by DSP whole-transcriptome analysis using B cell (CD20^+^) and T cell (CD3^+^) morphological windows (Figure 3H) (33). Consistent with the intratumoral profiling data, higher DZ signature expression correlated with lower abundance of CD3 area of interest (AOI) nuclei counts (Figure 3I). This association was further confirmed by multiplex IHC in 79 of the DLBCL cases, which confirmed that CD20^+^ tumor regions with elevated DZ signature expression displayed reduced CD3^+^ T cell density (Figure 3J).

These results establish a direct association between the DZ spatial transcriptional program and T cell exclusion in both reactive GCs and DLBCL.

### The GC DZ spatial signature is independent of AID-induced mutagenesis.

**Figure 4:**
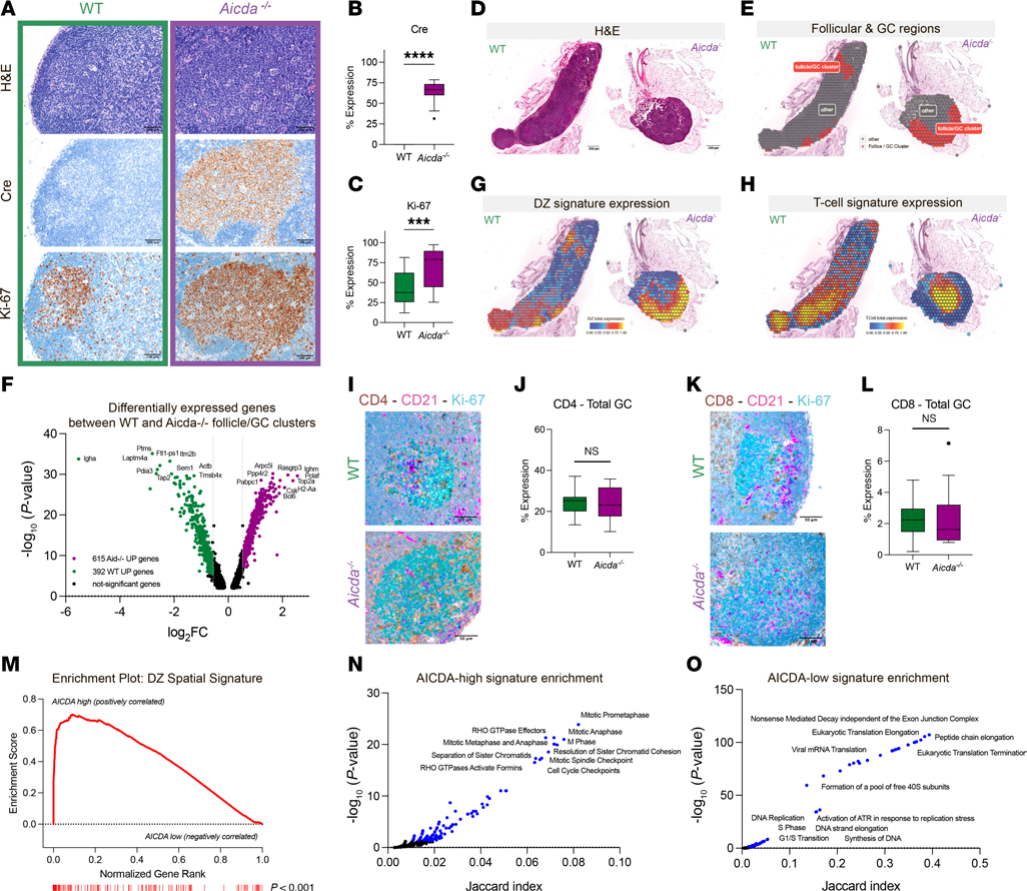
The spatial signature of DZ cells is independent of AICDA-related mutational processes. (**A**) Representative photomicrographs of H&E and IHC for Cre^+^ and Ki-67^+^ cells on mesenteric lymph nodes from WT and *Aicda^–/–^* mice. Original magnification, ×200. Scale bars: 100 μm. (**B** and **C**) Quantitative analyses of Cre^+^ (**B**) and Ki-67^+^ (**C**) cells in WT and *Aicda^–/–^* GCs (*n =* 20). Statistical analysis was assessed using a 2-tailed unpaired Mann-Whitney test. Values are shown as mean ± SEM; ***P < 0.001, ****P < 0.0001. (**D**) Representative photomicrographs of H&E-stained sections from WT and *Aicda^–/–^* mesenteric lymph nodes involved in the Visium spatial transcriptome experiment profiling. Original magnification, ×50. Scale bars: 250 μm. (**E**) Spatial visualization of WT and *Aicda^–/–^* follicle/GC clusters. (**F**) Volcano plot showing differentially expressed genes between WT cluster 4 and *Aicda^–/–^* clusters 1 and 3 (Wilcoxon’s rank sum test adjusted *P* values < 0.05, absolute log fold change > 0.025). (**G** and **H**) Spatial projection of DZ spatial signature (**G**) and T cell signature (**H**) total expression in WT and *Aicda^–/–^* samples. (**I**–**L**) Representative photomicrographs of triple IHC staining for DZ Ki-67^+^ (cyan signal), LZ CD21^+^ (pink signal), and CD4^+^ (**I**) or CD8^+^ cells (**K**) (brown signal) and quantitative analyses of the percentage of CD4^+^ (**J**) or CD8^+^ (**L**) T cells in WT and *Aicda^–/–^* GCs (*n =* 10 WT GCs; *n =* 10 *Aicda^–/–^* GCs). Original magnification, ×400. Scale bars: 50 μm. Statistical analysis: 2-tailed unpaired Mann-Whitney test. Mean ± SEM is shown. (**M**) Gene set enrichment analysis (GSEA) of DZ spatial signature in AICDA-high and AICDA-low DZ B cells. (**N**) Pathway enrichment of 257 *AICDA*-high signature genes using Reactome Pathway library. (**O**) Pathway enrichment of 127 *AICDA*-low signature genes using Reactome Pathway library.

AID plays a crucial role in DNA mutagenesis during immunoglobulin somatic hypermutation and contributes to the epigenetic heterogeneity of GC B cells (34). To determine whether AID activity is required to maintain the spatial DZ transcriptional signature, we analyzed GCs from Aicda-deficient (*Aicda^tm1(cre)Mnz/J^*) mice, in which the endogenous Aicda coding sequence is replaced with a Cre recombinase cassette (35). Cre expression was uniform across GC B cells in *Aicda^–/–^* mice, confirming effective knockout and providing a surrogate marker for Aicda locus inactivation (Figure 4, A and B). Spontaneous GCs in the mesenteric lymph nodes (mLNs) of *Aicda^–/–^* mice were larger and had an increased fraction of Ki-67^+^ proliferating cells compared with wild-type (WT) controls, consistent with prior findings that loss of AID results in impaired clonal selection and accumulation of proliferative centroblasts (Figure 4, A and C) (36).

To further investigate the impact of AID deficiency on the DZ signature, we conducted spatial transcriptomic profiling using Visium methodology on 1,950 microregions from mLNs of WT (*n =* 1,270) and *Aicda^–/–^* (*n* = 680) mice (Figure 4D). Unsupervised clustering identified 7 transcriptionally distinct regions in WT mLNs and 5 in *Aicda^–/–^* mLNs (Supplemental Figure 4, A–D, and Supplemental Table 5). Differential expression analysis of follicle/GC clusters revealed 1,007 differentially expressed genes (392 upregulated in WT and 615 in *Aicda^–/–^* GCs) (Figure 4, E and F, and Supplemental Table 6). As expected, *Igha* expression was elevated in WT GCs, whereas *Ighm* was upregulated in *Aicda^–/–^* GCs, consistent with the loss of immunoglobulin class-switch recombination in mutant cases.

Importantly, the *Aicda^–/–^* follicular/GC microregions were globally enriched for the DZ spatial signature compared with WT counterparts (Figure 4G, Supplemental Figure 4E, and Supplemental Table 7). This enrichment was accompanied by lower T cell signature expression (Figure 4H), which was further confirmed by IHC staining of CD4 and CD8 in total GCs and DZ/LZ regions (Figure 4, I–L, and Supplemental Figure 4, F and G). These results suggest that the DZ transcriptional program is independent of AID activity and that AID deficiency does not prevent T cell exclusion.

To investigate the transcriptional characteristics of the DZ signature independent of AID activity, we analyzed scRNA-Seq data from human GC DZ B cells (GSE139891), stratified into AICDA-high (AICDA expression > tertile 2) and AICDA-low (undetectable AICDA expression) groups (Supplemental Figure 4H). AICDA-high DZ cells exhibited significantly higher expression and enrichment of the DZ spatial signature compared with AICDA-low DZ cells (Figure 4M and Supplemental Table 7). Pathway analysis revealed that AICDA-high DZ B cells were enriched for genes involved in the G_2_/M cell cycle phase (Figure 4N, Supplemental Figure 4I, and Supplemental Table 8), whereas AICDA-low DZ B cells showed enrichment in DNA replication machinery genes and ATR-mediated replication stress pathways (Figure 4O and Supplemental Table 8).

These results suggest that ATR-mediated replication stress may contribute to the DZ transcriptional program and its associated T cell exclusion, independent of AICDA-induced mutagenesis.

### ATR inhibition rewires DZ programs, enabling a T cell–permissive environment.

Given the potential importance of ATR in maintaining DZ identity in an AID-independent manner, we further evaluated ATR-dependent pathways within the GC. DZ transcriptional profiling revealed an ATR-dependent activation of DDR and repair pathways. ATR is a key regulator of the cellular response to replication stress, playing a pivotal role in preserving chromatin organization and in restraining activation of the cGAS/STING pathway (37–39). ATR likely controls these key components of the DZ spatial signature (Supplemental Figure 5, A and B, and Supplemental Table 9).

Consistent with this, DZ B cells exhibited enrichment for chromatin remodeling and nuclear stability markers, including RAD51, γH2AX (phospho–S139-H2AX), phospho–S824-KAP1, SMARCA4 (BRG1), EZH2, and heterochromatin-associated markers H3K9me3 and HP1 (Supplemental Figure 5C). Furthermore, DZ B cells displayed features of chromatin compaction, as indicated by higher minimum DAPI intensity extracted from nuclear morphology and chromatin organization (NMCO) features in DAPI-labeled GCs (Supplemental Figure 5, D and E) (40). Heterochromatin features were predictive of DZ identity — as a random forest model trained on NMCO features accurately classified DZ and LZ B cells (Supplemental Figure 5F) — and correlated positively with DZ signature expression in DLBCLs (Supplemental Figure 5G).

Nuclear stability restricts cGAS/STING pathway activation, and we found that DNA and RNA sensing pathways were predominantly LZ restricted (Supplemental Figure 5H and Supplemental Table 9). Proximity ligation assay confirmed cGAS localization to the LZ, with minimal activity in the DZ (Supplemental Figure 5, I and J), supporting the notion that the DZ is an “immune-cold” environment, relatively resistant to inflammatory stimuli.

**Figure 5:**
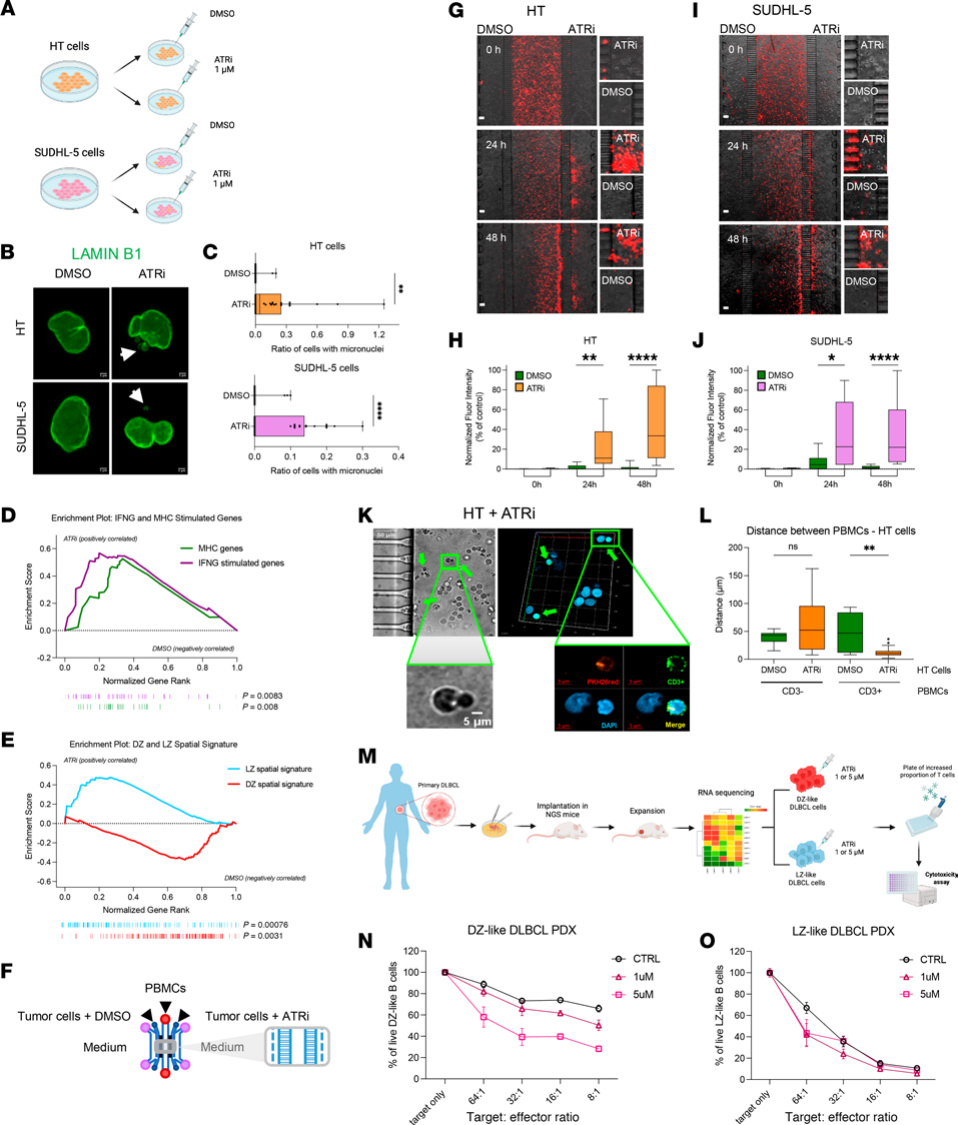
ATRi allows immune permeation of a DZ-like DLBCL in vitro. (**A**) Schematic of ATRi treatment (ceralasertib, AZD6738) in HT and SUDHL‑5 cells: cells treated with DMSO or AZD6738 (1 µM) for 48 hours. (**B**) Representative IF images of HT and SUDHL‑5 nuclei after 1 µM ATRi for 48 hours (green: lamin B1). Arrows indicate micronuclei. Scale bars: 5 µm. (**C**) Quantification of micronuclei formation (relative to IF analysis in **B**). (**D**) GSEA on ATRi and DMSO samples using the MHC and IFN‑γ signature. (**E**) GSEA on ATRi and DMSO samples using DZ and LZ spatial signatures. (**F**) Schematic of competitive microfluidic device. PKH26‑labeled PBMCs loaded into the central chamber; HT or SUDHL‑5 cells embedded in Matrigel with ATRi or DMSO and loaded in lateral chambers. (**G**–**J**) Visualization and quantification of red fluorescent PBMCs in HT (**G** and **H**) and SUDHL‑5 (**I** and **J**) chambers at 24 and 48 hours. (**G** and **I**) Scale bars: 125 µm. Mean ± SD from 3 replicates using PBMCs from different donors (*n* = 3). (**K**) Confocal microscopy of ATRi-treated DLBCL gel chamber at 48 hours. Arrows show interactions between CD3^+^ (green), PKH26^+^ (red) T cells and DAPI^+^ (blue) HT cells. Visible‑light image (left) and Z‑stack (right) shown. Scale bars: 50 µm (top left), 5 µm (bottom left and right). (**L**) Box plot showing distances between PBMCs and tumor cells in DMSO vs. ATRi chambers, measured in X, Y, Z coordinates in the microfluidic chip. (**M**) Schematic of experimental protocol. Primary tumor cells from DLBCL PDXs were selected based on spatial gene expression (DZ-like or LZ-like). Tumor cells pretreated with ATRi AZD6738 (1 or 5 µM) were used in T cell–mediated cytotoxicity assays. (**N**, **O**) Dose–response curves of T cell–mediated killing across indicated target/effector ratios for DZ-like (**N**) or LZ-like (**O**) DLBCL PDX-derived cells treated with 1 or 5 µM ATRi. Statistical analysis was assessed using a 2-tailed unpaired Mann-Whitney test (**C**, **H**, **J**, and **L**). Values are shown as mean ± SEM; *P < 0.05, **P < 0.01, ****P < 0.0001.

To investigate the role of ATR in maintaining the DZ transcriptional program, we treated two DZ-like lymphoma cell lines, HT and SUDHL-5 (Supplemental Figure 6A), with the ATR inhibitor (ATRi) ceralasertib (AZD6738) or DMSO control for 48 hours (Figure 5A and Supplemental Table 10). At 1 μM concentration, ATR inhibition did not impact cell viability (Supplemental Figure 6, B and C). ATRi treatment significantly increased micronuclei formation, a precursor to cGAS/STING activation (Figure 5, B and C) (41). Moreover, ATRi-induced transcriptional changes (Supplemental Table 11) included upregulation of interferon-stimulated genes and MHC-I/II transcripts, reversing the immune-evasive profile of DZ-like DLBCLs (Figure 5D and Supplemental Table 7). Notably, ATRi-treated DLBCL cells revealed a shift in transcriptional identity, with enrichment of the LZ spatial signature and suppression of the DZ spatial signature, suggesting that ATR activity reinforces the DZ transcriptional program (Figure 5E and Supplemental Table 7).

Next, to explore the potential impact of ATR inhibition on immune cell recruitment, we performed a competitive microfluidic assay (42, 43), coculturing peripheral blood mononuclear cells (PBMCs) from healthy donors with ATRi- or DMSO-treated HT and SUDHL-5 DZ-like DLBCL cells (Figure 5F). After 24 and 48 hours, PKH26-labeled PBMCs showed significantly increased infiltration into ATRi-treated chambers (Figure 5, G–J). Fluorescence microscopy revealed direct interactions between infiltrating CD3^+^ T cells and ATRi-treated DLBCL cells (Figure 5, K and L).

To evaluate the effect of enhanced T cell recruitment through ATR inhibition in a model representing the patient DLBCL immune context, we used primary cell lines derived from patient-derived tumor xenografts (PDXs). Among 22 PDX tumor samples, two were selected based on their DZ/LZ spatial signature expression: one DZ-like sample (high DZ, low LZ expression) and one LZ-like sample (low DZ, high LZ expression) (Figure 5M and Supplemental Methods). T cells were expanded by coculturing of PBMCs with corresponding irradiated (10 Gy) PDX cell lines, and the expanded T cells were subsequently harvested. T cell killing assays were performed across a range of effector-to-target ratios, in which LZ-like DLBCL cells displayed greater sensitivity to T cell–mediated killing compared with the DZ-like DLBCL cells. Importantly, ATRi pretreatment (1 μM and 5 μM) remarkably enhanced T cell killing of DZ-like DLBCL cells (Figure 5N), whereas LZ-like DLBCL cells (Figure 5O), which were already sensitive, showed no further improvement.

These results show that ATR inhibition reprograms DZ-like DLBCLs and attenuates their immune-silent state, facilitating increased T cell interaction and susceptibility to cytotoxicity.

### ATR inhibition promotes T cell infiltration in the GC microenvironment.

To validate these findings in vivo, we treated a total of 14 BALB/c mice with 25 mg/kg ATRi (*n* = 4 per time point) or vehicle control (*n* = 2 per time point) for either 2 or 5 consecutive days, and harvested mLNs the following day. After 5 days of treatment, ATRi-treated mice exhibited a significant increase in CD3^+^ T cell infiltration within chronic, spontaneously formed mLN GCs compared with vehicle-treated controls (Supplemental Figure 7A).

**Figure 6:**
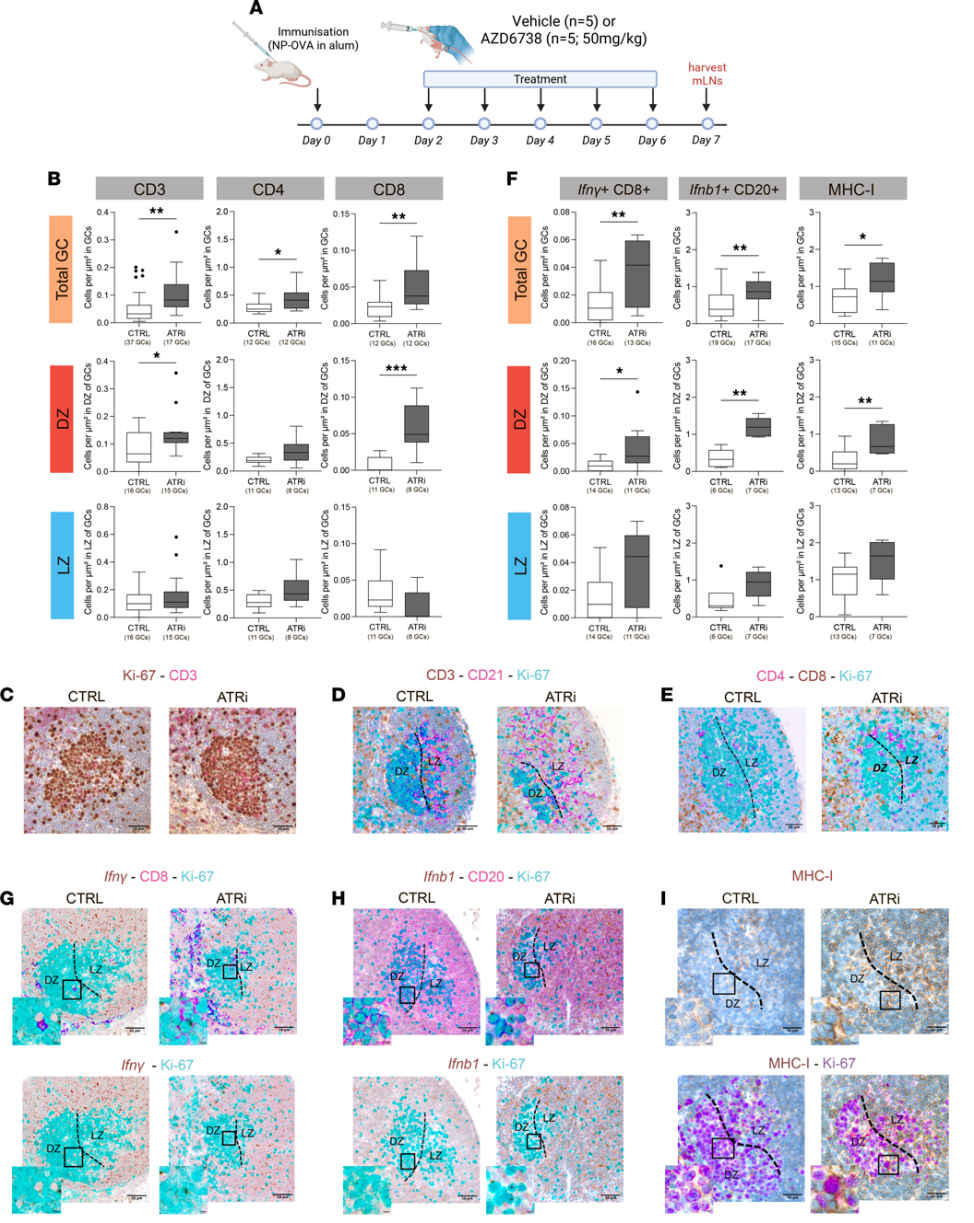
Functional impact of ATRi treatment on GC response. (**A**) Schematic representation of experimental protocol. In one experiment on ten C57BL/6J mice, immunization with NP-OVA in alum on day 0, followed by treatment with vehicle (*n* = 5) or AZD6738 (50 mg/kg, *n* = 5), was performed from day 2 to day 6. mLNs were harvested on day 7 for analysis. (**B**) Box plots showing quantitative analysis of CD3^+^, CD4^+^, and CD8^+^ T cell infiltration in indicated numbers of total GCs and DZ and LZ compartments in vehicle-treated (CTRL) versus ATRi-treated mice. (**C**) Representative photomicrographs of double-marker IHC for Ki-67^+^ (brown) and CD3^+^ (pink) cells in mLN GCs from vehicle-treated (CTRL) and ATRi-treated mice. Original magnification, ×400. Scale bars: 50 μm. (**D**) Combined IHC/IF staining for CD3^+^ (brown), CD21^+^ (pink), and Ki-67^+^ (cyan) cells in mLN GCs from vehicle-treated (CTRL) and ATRi-treated mice, showing spatial distribution of T cells in DZ and LZ compartments. Original magnification, ×400. Scale bars: 50 μm. (**E**) Combined IHC/IF staining for CD4^+^ (pink), CD8^+^ (brown), and Ki-67^+^ (cyan) cells in mLN GCs from vehicle-treated (CTRL) and ATRi-treated mice, illustrating phenotype of infiltrating T cells. Original magnification, ×400. Scale bars: 50 μm. (**F**) Box plots showing quantitative analysis of *Ifnγ*^+^CD8^+^ T cells, *Ifnb1*^+^CD20^+^ B cells, and MHC-I expression in indicated numbers of total GCs and DZ and LZ compartments in vehicle-treated (CTRL) versus ATRi-treated mice. (**G**) Representative images of combined mRNA ISH for *Ifnγ* (brown) and double-marker IHC for CD8 (pink) and Ki-67 (cyan) in mLN GCs from vehicle-treated (CTRL) and ATRi-treated mice, showing localization of activated CD8^+^ T cells. Original magnification, ×400 and ×630 (insets). Scale bars: 50 μm and 25 μm. (**H**) Representative images of combined mRNA ISH for *Ifnb1* (brown) and double-marker IHC for CD20 (pink) and Ki-67 (cyan) in vehicle-treated (CTRL) and ATRi-treated mice, highlighting induction of type I interferon response in the DZ. Original magnification, ×400 and ×630 (insets). Scale bars: 50 μm and 25 μm. (**I**) Representative IHC staining for MHC-I (brown) or MHC-I (brown) and Ki-67 (violet) in vehicle-treated (CTRL) and ATRi-treated mice, demonstrating increased MHC-I expression in the DZ in response to ATR inhibition. Original magnification, ×400 and ×630 (insets). Scale bars: 30 μm. Box plot statistical analysis: 2-tailed unpaired Mann-Whitney test. Mean ± SEM is shown; *P < 0.05, **P < 0.01, ***P < 0.001.

In a complementary experiment, C57BL/6 mice were immunized with the T cell–dependent antigen NP-OVA to induce GC formation (Figure 6A). Two days after immunization, mice (*n *= 5 per group) were treated with either 50 mg/kg ATRi or vehicle control for 5 consecutive days, and lymphoid tissues were harvested the following day. IHC staining of mLN sections for Ki-67 and CD3 revealed a higher number of CD3^+^ T cells per GC in ATRi-treated mice relative to controls (Figure 6, B and C, and Supplemental Figure 7, B and D).

Triple staining for CD3, CD21, and Ki-67 enabled spatial mapping of T cells within GC compartments, distinguishing the DZ (Ki-67^+^CD21^–^) from the LZ (CD21^+^). This analysis revealed that T cell infiltration occurred predominantly in the DZ, with no significant increase in the LZ (Figure 6D). Additional staining for CD4 and CD8 was performed to define the subset of infiltrating T cells, revealing increased numbers of both T cell subsets within GCs across both mouse models, with DZ-infiltrating T cells primarily composed of activated *Ifnγ*^+^ CD8^+^ cytotoxic T cells (Figure 6, B and E–G, and Supplemental Figure 7, C, E, and F).

Consistent with increased cytotoxic T cell infiltration, mRNA in situ hybridization (ISH) revealed induced *Ifnb1* expression in DZ B cells of ATRi-treated mice (Figure 6, F and H, and Supplemental Figure 7, C and G), indicating activation of a localized type I interferon response within the DZ microenvironment. Given the role of type I interferons in promoting antigen presentation, we assessed MHC class I protein expression by quantitative IHC. ATRi-treated mice exhibited a significant MHC-I enrichment in GC DZ regions, supporting a role for ATR in maintaining immune silencing by restricting CD8^+^ T cell recognition (Figure 6, F and I, and Supplemental Figure 7, C and H).

Together, these in vivo results indicate that ATR inhibition perturbs the immune-silent status of the GC DZ, inducing local type I interferon, enhanced MHC expression, and cytotoxic T cell recruitment.

## Discussion

In this study, we used high-resolution spatial transcriptomics to interrogate the immune architecture of the GC, with a particular focus on T cell positioning. Using single-cell-resolved WTX via the CosMx platform, we uncovered substantial spatial heterogeneity in T cell distribution across the GC. Most T cell subsets — including Tfh cells, Tregs, and memory CD8^+^ T cells — were preferentially enriched in the LZ, while CD4/CD8-DN and naive CD8^+^ T cells showed no zone-specific enrichment. These findings were confirmed at the protein level using hyperplexed immunofluorescence (MACSima), reinforcing the idea that the LZ serves as the primary site of T cell–B cell interaction within the GC, whereas the DZ remains largely T cell excluded.

To understand the molecular basis of this compartmental organization, we leveraged digital spatial profiling to derive a spatially accurate signature for the DZ and LZ regions based on transcriptional profiling. Our analysis revealed distinct transcriptional programs in these zones, with the DZ marked by upregulation of DNA damage response, cell cycle, and DNA replication stress pathways, while the LZ was enriched in immune signaling networks. These spatial gene expression programs, coupled with the exclusion of T cells from the DZ, suggested a functional compartmentalization that may be retained in GC-derived lymphomas.

DHIT and MHG B cell lymphomas — which exhibit DZ-like transcriptional features — tend to have reduced T cell infiltration (14, 44); however, the contribution of spatially defined DZ signatures to this immune-cold phenotype had not been directly examined. Using our GC-derived DZ signature, we stratified DLBCL cases and found that tumors with high DZ signature expression were consistently associated with low T cell abundance. These results suggest that DZ-like lymphomas may be intrinsically resistant to T cell–mediated immunotherapies, such as bispecific antibodies and CAR T cells, owing to an underlying biology of immune exclusion rooted in their spatial programming (45).

A key mechanistic insight from our work involves the DNA damage response kinase ATR. ATR is known to orchestrate the replication stress response in the GC DZ, preserving nuclear integrity during rapid proliferation (39). It has also been implicated in modulating immune responses in other cancers, where ATR inhibition can promote T cell recruitment and enhance immune activation (46, 47). However, its specific role in shaping the immune landscape of the GC — and of DZ-like lymphomas — has not been previously defined. In vivo, ATR inhibition disrupted the immune-silent character of the GC DZ, reinforcing its role as a key regulator of T cell exclusion. Treatment with ATR inhibitors led to selective infiltration of CD4^+^ and CD8^+^ T cells into the DZ, accompanied by activation of cytotoxic CD8^+^ T cells and induction of type I interferon signaling. Notably, this was associated with increased MHC-I expression on DZ B cells, suggesting that ATR normally functions to suppress antigen presentation and limit local immune activation (48). These findings support a model in which ATR activity reinforces the proliferative, immune-protected state of the DZ by actively suppressing cues that would otherwise recruit and engage T cells. By lifting this suppression, ATR inhibition reprograms the DZ into an immune-permissive environment, enhancing T cell access and effector function. This mechanism may extend to DZ-like DLBCLs, which similarly exhibit low T cell infiltration, and highlights ATR inhibition as a rational strategy to sensitize these tumors to T cell–based immunotherapies.

## Methods

### Sex as a biological variable

Our study examined both male and female human reactive tonsil samples and animals in a balanced manner. Animals of both sexes were randomly assigned to experimental groups to ensure unbiased representation. Throughout the course of the study, no significant sex-dependent differences were observed in any of the measured parameters.

### Collection and handling of human tissue samples

Formalin-fixed, paraffin-embedded (FFPE) samples of human tonsils with reactive follicular hyperplasia (*n* = 20) were selected from the archives of the Tumor Immunology Unit, University of Palermo, for in situ quantitative IHC and IF, mRNA in situ hybridization (ISH), and proximity ligation assay analyses. One FFPE lymph node tissue sample involved by DLBCL was collected from the archives of the Pathology Unit of the University of Brescia for quantitative IF analyses and digital spatial profiling of microregions from DLBCL-infiltrated areas.

### Murine models

*Aicda^tm1(cre)Mnz/J^* (JAX:007770) and wild-type C57BL6/J mice were obtained from The Jackson Laboratory. Animals were regularly monitored by veterinary personnel throughout the duration of the experiments. Mice were checked at least 3 times a week for signs of illness and any reduction or impairment in motility. The experimental mice were followed until they reached 28–32 weeks of age. At this point they were euthanized to collect mesenteric lymph nodes for histopathological, immunolocalization, and spatial transcriptomic analyses.

Male BALB/c mice (InVivos Singapore) were randomly assigned to vehicle control (*n* = 2) or ATR inhibitor (ATRi) treatment (*n* = 4) groups. AZD6738, an ATRi, was dissolved at 2.5 mg/mL in a vehicle solution containing 10% DMSO, 40% propylene glycol, and 50% deionized sterile water. Mice in the treatment group received 25 mg/kg AZD6738 by oral gavage daily for either 2 or 5 consecutive days. Tissues were harvested on day 3 or day 6, respectively. Control mice received an equivalent volume of vehicle solution.

In a separate experiment, 6-week-old male C57BL/6J mice (The Jackson Laboratory) were immunized intraperitoneally with 4-hydroxy-3-nitrophenylacetyl–conjugated ovalbumin (NP-OVA) adsorbed on alum. Two days after immunization, mice (*n* = 5 per group) received 50 mg/kg AZD6738 daily by oral gavage for 5 consecutive days. Spleens and mesenteric lymph nodes were harvested 24 hours after the final dose. All harvested tissues were immediately fixed in cold 10% neutral-buffered formalin for 12 hours, followed by standard paraffin embedding procedures.

### PDX-derived primary cell lines and in vitro killing assay

Twenty-two primary DLBCL tumor samples were implanted subcutaneously into NSG-S mice (The Jackson Laboratory) to generate PDX models (49). Once tumors reached the appropriate size, they were excised, enzymatically and mechanically digested, and cultured in RPMI 1640 medium supplemented with 10% fetal bovine serum (FBS) and IL-2. After stabilization in vitro, samples were ranked based on the expression of the DZ and LZ signatures, from which 1 DZ-like and 1 LZ-like sample were used for downstream experiments. T cells were expanded by coculturing of PBMCs with 10-Gy-irradiated DLBCL tumor cells in RPMI 1640 supplemented with 10% FBS, IL-2, IL-7, and IL-15. Expanded T cells were harvested and used in cytotoxicity assays. PDX-derived DLBCL cells were treated with the ATRi ceralasertib (AZD6738) at 2 concentrations (1 μM and 5 μM) or DMSO for 4 days. After treatment, 1 × 10^5^ tumor cells were plated per well in 96-well plates. Expanded T cells were added at varying effector-to-target ratios. After 72 hours of coculture, cells were stained with anti-CD3, anti-CD19, and DAPI, and analyzed by flow cytometry to quantify T cell–mediated tumor killing.

### DLBCL cell culture and treatment

HT and SUDHL-5 cell lines were obtained from the ATCC and selected based on the high expression of the DZ spatial signature according to the 23Q2 DepMap gene expression dataset. HT and SUDHL-5 cells were cultured in RPMI medium supplemented with 1% glutamine, 10% FBS, and penicillin-streptomycin. Suspension cultures were maintained in flasks in 5% CO_2_, at 37°C. The cells were treated for 48 hours with clinical-grade ATRi ceralasertib (AZD6738, S7693 Selleckchem; 1 μM) and DMSO for the untreated control. Cell lines were obtained from the ATCC and tested for mycoplasma contamination, with negative results. Additional information on micronuclei analysis, RNA extraction, quantitative PCR, and RNA-Seq is available in Supplemental Methods.

### Competitive migration assay in microfluidic devices

Microfluidic devices were fabricated in polydimethylsiloxane (PDMS), a biocompatible silicon elastomer, as previously reported (50). The device allowed visualization of preferential PBMC migration toward ATRi- or DMSO-treated HT and SUDHL-5 cells embedded in 3D hydrogels. Additional information on cell loading, labeling, and quantitative analysis is available in Supplemental Methods.

### Hyperplexed MACSima analysis

Multiplex IF analyses were performed on FFPE tonsil sections using the MACSima platform (Miltenyi Biotec) (51). Tissue sections were processed and stained in a fully automated manner, with sequential immunolabeling, imaging, and quantification analyses. Further details on the protocol and antibody panel are provided in Supplemental Methods.

### Quantitative ISH and immunolocalization analyses

Single and multiplexed IHC and IF stainings and in situ mRNA ISH were performed on FFPE human or murine tissue sections as previously described (52). The detailed protocol and antibodies adopted are included in Supplemental Methods. IHC-stained slides were digitalized using an Aperio CS2 digital slide scanner (Leica Microsystems), and IF-stained slides were analyzed and imaged under a Zeiss Axioscope-A1 equipped with wide-field fluorescence module and Axiocam 503 Color camera (Zeiss). Quantitative analyses were performed using HALO image analysis software for cell segmentation and signal quantification (v3.2.1851.229, Indica Labs) as detailed in Supplemental Methods.

### In situ proximity ligation assay

Proximity ligation assay (PLA) was conducted on FFPE sections from human tonsil samples using the NaveniFlex Tissue MR Red kit following the manufacturer’s instructions (Navinci Diagnostics). The antibodies adopted for test and control PLA assays are listed in Supplemental Methods. Quantitative analysis of PLA signals was performed through HALO image analysis software (v3.2.1851.229, Indica Labs) as detailed in Supplemental Methods.

### In situ transcriptional analyses

Ten DZ and LZ regions of interest (ROIs) within morphologically normal FFPE human tonsillar GCs were profiled using the GeoMx Digital Spatial Profiler (NanoString Technologies). Tissue sections were stained with CD271/NGFR and CD20, as described in our previous work (21). The selected and segmented DZ and LZ ROIs were analyzed for the expression of 1,824 curated genes included in the Cancer Transcriptome Atlas (CTA) panel (NanoString).

Spatial transcriptomics of DLBCL tissues was performed using the GeoMx Whole Transcriptome Atlas (WTA) kit (NanoString), according to the manufacturer’s protocol. FFPE tissue sections were processed on the Leica Bond Max automated system, followed by ISH. Sections were stained with CD3 and CD20 to visualize T and B cells, respectively. ROIs were selected based on pathologist recommendations and analyzed using the GeoMx DSP platform. CD3^+^ and CD20^+^ signal masks were generated and submitted for downstream sequencing and data processing. Full details of the DSP workflow, including ROI selection, library preparation, sequencing, and data normalization, are provided in our previous work (33).

Additionally, 11 ROIs were selected from a DLBCL-infiltrated FFPE lymph node tissue sample based on CD20 and CD3 staining and profiled using the same CTA panel. Further information on DSP data analysis is provided in the “Statistical and bioinformatics analyses” section below, and in Supplemental Methods.

For murine tissue analysis, spatial transcriptomics was performed on FFPE mesenteric lymph nodes using the 10x Genomics Visium platform, following the manufacturer’s instructions. Details regarding library preparation, sequencing, and data analysis for the Visium experiment are available in Supplemental Methods.

### CosMx Whole Transcriptome Atlas

#### Processing of data.

CosMx SMI Whole Transcriptome (WTX) was shared with us by Bruker Spatial Biology as a Seurat object. The quality control (QC) filters were applied, retaining cells with nFeature_RNA counts less than 3,000 and nCount_RNA greater than 400. The filtered dataset was then converted to the AnnData format for downstream analysis with Python-based Scanpy (https://scanpy.readthedocs.io/en/stable/). The final dataset had 132,676 cells across 18,935 targets.

#### Data normalization.

The top 5,000 highly variable genes were identified with the scanpy.experimental.pp.highly_variable_genes function, which uses Pearson residuals as basis for selection. The data were then log-transformed and scaled. Scaled values were subjected to principal component analysis (PCA) for linear dimension reduction. A nearest-neighbor network was created based on Euclidean distances between cells in a multidimensional PC space (the first 50 PCs were used). For visualization, the uniform manifold approximation and projection (UMAP) technique was used.

#### Annotation of germinal centers.

Germinal centers (GCs) were identified based on expression of 7 GC B cell markers (BCL6, AICDA, CD38, LMO2, MEF2B, PIM1, ST6GAL1, and EZH2) and DAPI intensities derived from IF images in the CosMx experiment. The expression levels of GC B cell markers and DAPI intensities were visualized in Napari, (https://napari.org/stable/) where GC regions were manually annotated. Annotation was based on circular clusters exhibiting high GC B cell marker expression and reduced DAPI signal relative to the surrounding cells. In total, nine GCs were identified.

#### DZ and LZ segmentation.

The 169-gene DZ signature and the 201-gene LZ signature were scored in the dataset and visualized. It was observed that the GCs were polarized based on the DZ and LZ signatures. To determine the DZ or LZ status of each individual cell, we subtracted the LZ signature score from the DZ signature score to form a combined DZ-LZ score. A highly positive DZ-LZ score (> 0.3) corresponds to a DZ phenotype (termed DZ cells), and a highly negative DZ-LZ score (< –0.3) corresponds to an LZ phenotype (termed LZ cells). Remaining cells were allocated to an intermediate phenotype.

To identify putative DZ and LZ regions within each GC, an iterative graph-based cell neighbor analysis was performed. Briefly, a spatial graph G was constructed to represent cellular relationships. Each node in G corresponded to a cell, with attributes including *x* and *y* coordinates representing its spatial location and a phenotype label derived from previous DZ/LZ classification. Edges were created between nodes based on Euclidean distance, capturing the local neighbors of each cell. The pairwise Euclidean distances between all cells were precomputed using the SciPy function cdist, (https://docs.scipy.org/doc/scipy/reference/generated/scipy.spatial.distance.cdist.html) generating a distance matrix for all cell pairs, allowing for efficient identification of neighboring cells within a fixed spatial threshold.

In each iteration, neighbors of each cell within the GC will be checked and reannotated based on a predetermined fraction of dissimilar neighbors. Cells with an intermediate phenotype were reannotated based on the most common phenotype of their immediate neighbors. The iterative process was repeated until no further changes to cell annotations were made. To maintain an accurate representation of the DZ and LZ regions, the DZ region was required to contain at least 70% DZ cells, and similarly, the LZ region was required to contain at least 70% LZ cells. Once this condition was met, the boundary between the two regions was established and retained for subsequent downstream spatial analyses.

#### Label transfer by MaxFuse.

Cell type annotation was achieved by label transfer from a well-annotated reference scRNA-Seq HCATonsil dataset. Label transfer was done using MaxFuse (https://github.com/shuxiaoc/maxfuse) which could integrate data across different modalities such as spatial and suspension single-cell RNA datasets, through cross-modality matching and iterative smoothed embedding. To ensure robust label transfer, the top 5,000 highly variable genes common to both the scRNA-Seq and CosMx WTX spatial datasets were identified and used as matched pairs. After label transfer, spatial distributions of subpopulations of T cells were analyzed within the DZ and LZ regions of the GCs.

#### Spatial distribution of T cells.

Using the DZ and LZ boundary line as reference, we assessed the distribution of T cell subpopulations from –100 μm (DZ) to 100 μm (LZ) from the reference line. Cells were binned into 10-μm increments based on their distance from the boundary, and the mean enrichment level of each T cell subtype for each bin was computed, providing a continuous spatial profile of T cell subtype enrichment across the boundary. Linear regression coefficient of the observed slope line was used as a measure of spatial distribution pattern across the boundary. To determine whether the observed enrichment trend was significantly different from a random distribution, we performed 10,000 Monte Carlo simulations in which cell positions of each subtype were randomly shuffled while ensuring that its total cell count remained fixed. For each iteration, a new linear regression was fitted to the randomized enrichment-distance relationship, generating a null distribution of slopes, under the assumption of complete spatial randomness. The Monte Carlo confidence envelope was plotted as a shaded region, representing the range of slopes expected under random conditions. A 95% CI was constructed from these simulations: (a) If the observed slope fell above the upper 95% CI, enrichment of the T cell subtype was significantly increasing with distance from DZ into LZ. (b) If the observed slope fell below the lower 95% CI, enrichment of the T cell subtype was significantly decreasing with distance from DZ into LZ. (c) If the observed slope remained within the Monte Carlo envelope, enrichment variation was consistent with randomness.

### Computational pipelines to characterize the chromatin states of DZ and LZ cells

Computational analyses were conducted on digital images derived from 2 datasets: 15 manually identified GCs from AID/CD3 immunofluorescence–stained tissues and 11 DSP-profiled DLBCL ROIs stained for CD20 and CD3. DZ and LZ B cells were classified based on nuclear chrometric features by application of a random forest classifier. The relationship between chrometric states and the DZ signature was evaluated in the selected in situ transcriptionally profiled microregions. Additional information on pipeline implementation, image processing, and statistical analyses is available in Supplemental Methods.

### Statistics

The spatial DZ and LZ signatures were obtained by comparison of the gene expression of paired human tonsil DZ and LZ GC ROIs (*n* = 10) profiled by NanoString digital spatial profiling as previously reported (21). Upregulated/downregulated genes were selected using the limma moderated statistic (Benjamini-Hochberg adjusted *P* values < 0.05) (53). The Reactome Pathway library was used for pathway enrichment analysis (ReactomePA R package) (54). The Euclidean distance and the Ward.D2 method were used for unsupervised clustering. The SpatialDecon algorithm (55) was adopted to estimate cell fractions on DSP data, while the xCell algorithm (32) was used to estimate selected immune and stromal cell type enrichment scores on bulk RNA-Seq samples. Additional information on unsupervised hierarchical clustering, pathway and gene set enrichment analyses, DZ/LZ scRNA-Seq analysis, adopted DLBCL gene expression datasets, immune and stromal deconvolution, survival analysis on DLBCL datasets, and Visium spatial transcriptomics analysis is available in Supplemental Methods.

### Study approval

The human samples for the PDX model were collected and handled according to the Helsinki Declaration, and the study was approved by the University of Palermo Ethical Review Board (approval numbers 09/2018 and 04/2023). Use of patient samples from National University Hospital, Singapore, was approved by National Healthcare Group Domain Specific Review Board (NHG DSRB) (reference 2015/00176; title: SINGAPORE Lymphoproliferative Disease STUDY).

All animal experiments were conducted in compliance with institutional and national ethical guidelines. Studies involving *Aicda^tm1(cre)Mnz/J^* mice were approved by the Animal Welfare Organization (OPBA) of Palermo, Italy, and by the Italian Ministry of Health, and were carried out in accordance with Italian legislation (D.lgs 26/2014; authorization 495/2020-PR).

Experiments involving ATRi-treated animals were approved by IACUC (reference 181412 and 231766 from Biological Resource Centre, A*STAR).

### Data availability

All data generated in the present work have been made publicly available. The DSP data relative to 11 profiled DLBCL ROIs are reported in Supplemental Table 4. The human bulk RNA-Seq FASTQ files were deposited in the NCBI’s Sequence Read Archive under accession code PRJNA1082634, while the read counts are reported in Supplemental Table 10. The raw and processed data of Visium spatial transcriptomics were deposited in the NCBI’s Gene Expression Omnibus (GEO) database under accession code GSE260998. The DSP RNA-Seq data profiled on tonsil GC DZ and LZ ROIs are publicly available (21). Values for all data points in graphs are reported in the Supporting Data Values file.

## Author contributions

VC, G Bertolazzi and ASYC are co–first authors, with VC listed first as she initiated the project. VC, G Bertolazzi, ASYC, SC, ADJ, and CT designed the research studies. VC, G Bertolazzi, ASYC, G Medico, G Bastianello, G Morello, DP, CL, HL, G Shenoy, FM, MVR, FN, LB, A De Ninno, IC, SV, GC, DV, BB, M Liu, M Lakshmanan, MSNO, ZB, TS, and EM conducted the experiments and analyzed data. PWJ, G Schiavoni, SS, KPL, GV, MPC, SB, GI, LC, MF, SC, ADJ, and CT supervised the experiments and acquired data. SS, LC, SC, ADJ, and CT acquired funding. VC, G Bertolazzi, ASYC, G Medico, G Bastianello, G Morello, DP, CL, HL, G Shenoy, PWJ, G Schiavoni, FM, SP, MVR, FN, FP, SV, SS, A Di Napoli, SL, LL, AJMF, BB, GV, MPC, SB, GI, LC, MP, RZ, EM, JB, FF, MF, SC, ADJ, and CT discussed and interpreted the results. VC, G Bertolazzi, ASYC, G Medico, SC, ADJ, and CT wrote the manuscript. CT, ADJ, and SC are co–last authors, with CT listed last as he conceptualized the study.

## Supplementary Material

Supplemental data

Supplemental table 1

Supplemental table 2

Supplemental table 3

Supplemental table 4

Supplemental table 5

Supplemental table 6

Supplemental table 7

Supplemental table 8

Supplemental table 9

Supplemental table 10

Supplemental table 11

Supporting data values

## Version 1 07/17/2025

In-Press Preview

## Version 2 09/16/2025

Electronic publication
