# Supplementary material for: Aggressive B cell lymphomas retain ATR-dependent determinants of T cell exclusion from the germinal center dark zone
Source: J Clin Invest. 2025 Jul 17;135(18):e187371. doi: 10.1172/JCI187371 (PMC12435852; doi:10.1172/JCI187371)
Supplement: Supplemental data [file jci-135-187371-s244.pdf]

SUPPLEMENTARY INFORMATIONS

Aggressive B-cell lymphomas retain ATR-dependent determinants of T-cell exclusion from the Germinal Center Dark Zone

Cancila *et al.*

SUPPLEMENTARY METHODS

SUPPLEMENTARY REFERENCES

SUPPLEMENTARY FIGURES 1 – 7

SUPPLEMENTARY METHODS

Hyperplexed MACSima imaging and quantitative analysis

Multiplex immunofluorescence analyses on formalin-fixed, paraffin-embedded (FFPE) sections on human tonsil were performed using the MACSima Imaging Platform (Miltenyi Biotec) with the following primary antibodies at 1:50 dilution and 10-minute incubation: CD3 (clone REA1151, FITC, catalog no. 130-120-267, REAfinity™), CD4 (clone REA1307, PE, catalog no. 130-127-906, REAfinity™), CD45RO (clone REA611, PE, catalog no. 130-113-559, REAfinity™), CD8α (clone REA1024, FITC, catalog no. 130-117-200, REAfinity™), CD279 (PD-1, clone REA1165, PE, catalog no. 130-120-382, REAfinity™), CD57 (clone REA769, PE, catalog no. 130-111-810, REAfinity™), Ki-67 (clone REAL1047, FITC, catalog no. 130-127-837, REAfinity™), CD271 (NGFR, clone REAL709, APC, catalog no. 130-125-057, REAfinity™). T cell subtypes were identified based on the following surface markers: TFH cells: $CD3^+$ $CD4^+$ $CD57^+$ and $CD3^+$ $CD4^+$ $CD279^+$; Memory $CD4^+$ T cells: $CD3^+$ $CD4^+$ $CD45RO^+$; Memory $CD8^+$ T cells: $CD3^+$ $CD8^+$ $CD45RO^+$; Naïve $CD4^+$ T cells: $CD3^+$ $CD4^+$ $CD45RO^-$; Naïve $CD8^+$ T cells: $CD3^+$ $CD8^+$ $CD45RO^-$; Double-negative (DN) T cells: $CD3^+$ $CD4^-$ $CD8^-$. Sections, 3 µm thick, were deparaffinized, rehydrated, and subjected to heat-induced epitope retrieval using a Preheat PT- Module with TEC-buffer (pH9; Trizma Base, EDTA, Sodium citrate tribasic dihydrate) at 85°C, followed by incubation at 98°C for 20 minutes and cooling at 85°C for an additional 20 minutes. After washing with MACSima Running Buffer, sections were mounted on MACSwell Imaging Frames (Four-well format), and a DAPI pre-staining was performed prior to initiating the automated imaging protocol. The MACSima Imaging System integrates liquid handling with widefield microscopy to enable cyclic immunofluorescence (IF), including automated staining, washing, imaging, and signal erasure steps via photobleaching. For REAfinity antibodies, signal erasure was achieved via LED- based photobleaching: blue LED (2 W/cm², 2 min), green LED (0.4 W/cm², 2 min), or red LED (1 W/cm², 6 min), resulting in >90% reduction in FITC, PE, or APC signal. Imaging was performed with epifluorescence optics using three objectives (20× NA 0.75; long-working-distance 20× NA 0.45; and 2× NA 0.1). Excitation was provided by custom LED sources (infrared, red, green, blue, UV) and filtered to narrow the excitation spectrum. Images were captured with a monochromatic sCMOS camera. Autofocus was achieved by hardware control and DAPI-based image optimization. The MACSima image processing pipeline included HDR image generation, pixel correction, optical profile normalization, and subtraction of pre-stain autofluorescence. Processed image datasets were visualized and exported using MACS iQ View software (Miltenyi). Quantitative image analysis was performed using the HighPlex FL v4.2.3 segmentation-based algorithm within HALO software (Indica Labs). Analyses were focused on nine GCs, where DZ and LZ regions were manually segmented based on the expression of Ki-67 and CD271.

In situ mRNA hybridization and quantitative immunolocalization analyses

Four-micrometer–thick human and mouse tissue sections were deparaffinized, rehydrated, and unmasked using Novocastra Epitope Retrieval Solutions at pH6 or pH9 (Leica Biosystems) in a thermostatic bath at 98°C for 30 minutes. The sections were then brought to room temperature and washed in PBS. After neutralization of the endogenous peroxidase with 3% H_2O_2 and Fc blocking with 0.4% casein in PBS (Leica Biosystems), the sections were incubated with primary antibodies. For multiple-marker immunostaining, sections were underwent sequential rounds of single-marker immunostaining with the binding of primary antibodies revealed using specific secondary antibodies conjugated with different fluorophores or enzymes.

The following primary antibodies were used for immunohistochemistry (IHC) and IF on mouse and/or human tissues: rabbit monoclonal p75 NGFR (clone EP1039Y, 1:200 pH6, catalog no. ab52987, Abcam), rabbit polyclonal Ki-67 (1:1000 pH6, catalog no. ab15580, Abcam), mouse monoclonal CD8 (clone 4B11, 1:50 pH9, catalog no. NCL-L-CD8-4B11, Leica Biosystems), rabbit monoclonal CD4 (clone EPR6855, 1:500 pH9, catalog no. ab133616, Abcam), rabbit monoclonal AICDA (clone EPR23436-45, 1:2000 pH9, catalog no. ab269454, Abcam), rabbit polyclonal (p)gHistone 2AX (1:1000 pH6, catalog no. ab11174, Abcam), rabbit monoclonal Rad51 (clone EPR4030(3), 1:200

pH9, catalog no. ab133534, Abcam), rabbit monoclonal pKap1 (clone BL-246-7B5, 1:100 pH9, catalog no. ab243870, Abcam), mouse monoclonal SMARCA4/Brg1 (clone G-7, 1:50 pH9, catalog no. sc-17796, Santa Cruz Biotechnology), mouse monoclonal EZH2 (clone 6A10, ready to use pH9, catalog no. PA0575, Leica Biosystems), rabbit monoclonal CD21 (clone SP186, 1:100 pH9, catalog no. ab240987, Abcam), rabbit polyclonal CD3 (1:100 pH9, catalog no. ab5690 Abcam), mouse monoclonal TCRδ (clone H-41, 1:50 pH9, catalog no. sc-100289, Santa Cruz Biotechnology), rabbit polyclonal Histone H3 (tri methyl K9) (1:400 pH6, catalog no. ab8898, Abcam), mouse monoclonal HP1α (clone 2HP-1H5, 1:500 ph9, catalog no. MAB3584, Merck Millipore), rat monoclonal CD3 (clone CD3-12, 1:100 pH9, catalog no. ab11089 Abcam), mouse monoclonal CD68 (clone 514H12, ready to use pH9, catalog no. PA0273, Leica Biosystems), rabbit monoclonal Plk1 (clone 208G4, 1:50 pH9, catalog no. #4513, Cell Signaling Technology), rabbit monoclonal Egr1 (clone EPR15916, 1:100 pH9, catalog no. ab194357, Abcam), rabbit monoclonal Cre (clone D7L7L, 1:100 pH9, catalog no. #15036, Cell Signaling Technology), rabbit monoclonal CD4 (clone D7D2Z, 1:50 pH9, catalog no. #25229, Cell Signaling Technology), rabbit monoclonal CD8α (clone D4W2Z, 1:400 pH9, catalog no. #98941, Cell Signaling Technology), rabbit monoclonal MHC-I (clone E8E7N, 1:250 pH6, catalog no. #76828, Cell Signaling Technology), rat monoclonal CD20 (clone GOT214A, 1:50 pH9, CNIO Monoclonal Antibodies Unit). Immunohistochemistry (IHC) staining was developed using the Novolink Polymer Detection Systems (Leica Biosystems) or IgG (H&L)-specific secondary antibodies (Life Technologies, 1:500) and DAB (3,3′-Diaminobenzidine, Novocastra) as substrate chromogen.

Double IHC staining was performed using either the SignalStain Boost IHC Detection Reagent alkaline phosphatase-conjugated (anti-rabbit, produced in horse; Cell Signaling Technology) or ImmPRESS AP Polymer Detection Reagent (anti-rat, produced in goat; Vector Laboratories), with Vulcan Fast Red as the substrate chromogen. Alternatively, SignalStain Boost IHC Detection Reagent horseradish peroxidase (HRP)-conjugated (anti-mouse, produced in goat, Cell Signaling Technology) was used in combination with PolyDetector HRP Green chromogen. All procedures were carried out according to the manufacturers' instructions.

For IF multiplex stainings, anti-mouse and anti-rabbit secondary antibodies conjugated with Alexa Fluor 488 and 568, respectively, were used. DAPI (4′,6-diamidin-2-fenilindolo) was utilized to counterstain the nuclei.

Hybridization with human *IFNγ* (NM_000619.2), mouse *Ifnb1* (NM_010510.1), and *Ifnγ* (NM_008337.3) probes was performed using RNAscope 2.5 HD Detection Reagent-BROWN (Advanced Cell Diagnostic) in accordance with the manufacturer's protocol.

Slide digitalization was conducted using an Aperio CS2 digital scanner (Leica Biosystems) with the ImageScope software (Aperio ImageScope version 12.3.2.8013, Leica Biosystems).

Quantitative analyses of IHC stainings and *in situ* mRNA were performed by calculating the average percentage of positive cells in twenty non-overlapping human GCs at medium-power magnification (x200) using the HALO image analysis software (v3.2.1851.229, Indica Labs) and the output was expressed as the "percentage of positive cells".

In murine mesenteric lymph nodes, the total number of GCs was also quantified and, when polarization was clearly identifiable, DZ and LZ areas were segmented and analyzed separately.

Spatial Analyses on quantitative IHC and IF data

To analyze the infiltration of specific cell populations in different regions of interest within the tissue and examine the spatial relationships between different cell populations using the Nearest Neighbor analysis tool, the HALO software (v3.2.1851.229, Indica Labs) was instructed to generate spatial maps based on the markers of interest. The Mann-Whitney-Wilcoxon test was applied to compare positive cell fractions among conditions.

HALO *Infiltration analysis* was performed on dual ISH-IHC for *IFNγ* and AID. Within each GCs, a 100 μm wide (-50 to +50) LZ/DZ interface was defined, and the density of *IFNγ*+ infiltrating cells was calculated in each region.
HALO Nearest Neighbor analysis was applied to determine the mean distance and the number of unique neighbors between two cell populations of interest, specifically PLK1 or EGR1 with CD4 and CD8 T cells.
For quantitative estimation of cell-cell repulsion, we developed an ad hoc computational approach based on resampling (1). This approach was applied to digital images from GCs stained by multiplexed IHC and IF. We calculated the distances of each AID-positive cell from the nearest cell of the population of interest (i.e., CD3 and CD68). The observed nearest distance distributions were compared with the nearest distance distribution calculated on randomized cell spatial projections. The Wilcoxon-Mann-Whitney test was used to compare the nearest neighbor distance distributions. The cumulative density function (CDF) was used for graphical representation.

In situ Proximity Ligation Assay
Proximity ligation assay (PLA) was performed with NaveniFlex Tissue MR Red kit (Navinci Diagnostics). The following primary antibodies were used: rabbit monoclonal cGAS (clone D1D3G, 1:100 pH9, catalog no. #15102, Cell Signaling Technology), mouse monoclonal DNA double stranded (clone AE-2, 1:100 pH9, catalog no. #MAB1293, Merck Millipore). After primary antibody incubation, the secondary antibodies oligonucleotides-conjugated were added to the specimens.
The control experiments were performed for each combination by omitting one of the primary antibodies. The PLA signals were quantified in ten non-overlapping GCs at medium magnification (x200) using the HALO image analysis software (v3.2.1851.229, Indica Labs).

Computational pipelines to characterize the chromatin states of DZ and LZ cells
To characterize the chromatin states of cells the pipeline first range-normalizes the individual images to correct for differences in the illumination of the individual images. Thereby, the normalized intensity I(x,y) of each image I were computed as

$$I(x,y) = \frac{I'(x,y) - Q_1(I)}{Q_{99.8}(I) - Q_1(I)},$$

where I'(x,y) is the raw intensity of the DAPI image of the pixel at position (x,y) and Q_p (I) is the p-th percentile of the intensity distribution of the image within the selected microregion or GC.
Next, individual nuclei were segmented using a pre-trained StarDist model from(2) with the proposed default parameters. Finally, the nuclei masks and the normalized DAPI images were used to extract a number of features for each nucleus jointly characterizing their nuclear morphology and chromatin organization patterns, i.e. their chromatin states. To this end, we used the python package chrometrics from (3).
To identify the cell-types of the individual cells within the GCs, we first generated approximate cellular masks following an approach proposed in (4). The method obtains the cellular masks by expanding the nuclear boundaries of the individual cells for a maximum of 1.6 microns or until the boundary was seen to overlap with those of another cell. Next, we identified cells that were positive or negative for the DZ marker AID and CD3 by analyzing the cellular mean intensity of these markers in the IF images for each cell. Cells were identified as positive for the respective marker if their mean intensity exceeded an image-specific threshold. The threshold was calculated as the average of the means of a two-component Gaussian mixture model fitted to the mean intensity values. The accuracy of the classification was visually validated. Finally, we identified dark-zone B-cells (i.e. cells positive for

dark-zone B-cell marker AID and negative for CD3), light-zone B-cells (i.e. cells both negative for AID and CD3) and T-cells (negative for AID and positive for CD3).

To assess whether the chromatin states of the B-cells in the LZ differ from those in the DZ, we first selected 9,197 LZ B-cells uniformly at random from all LZ B-cells of across all of the 11 selected GCs (n = 16,182). This was done to match the total number of DZ B-cells across all GCs and thus obtain a balanced subsample. Each of the B-cells was described by 60 chrometric features which we selected as a subset of the 177 measured features by removing all features correlated with an absolute Pearson correlation of above 0.8 when considering all cells in the data set.

We next evaluated the separability of the LZ and DZ B-cells based on their chromatin states characterized by the chrometric features by training a random forest classifier to classify the two types of B-cells given the chrometric states. A Random Forest classifier is a tree-based classification algorithm that fits an ensemble of decision trees and aggregates the output of the individual trees to enable robust classification of samples into a predefined set of classes (5). We evaluated the performance of the class separability LZ and DZ B-cells by assessing the average balanced accuracy (6) of the classifier in a 10-fold stratified cross-validation setup. To this end, we applied the default parameterization of the scikit-learn (7) python package that we used to perform the analysis. The classifier was able to distinguish between the two types of B-cells given the chrometric features with an average accuracy of 0.6256 (+/- 0.0419). Thereby, the minimum intensity was the most discriminative feature as measured by the Gini importance. In alignment with this result, we found the mean of this chrometric feature to differ significantly (p-values < 10^{-125}, Welch t-test).

To correlate the chromatin state of cells in the selected microregions with the measured DZ expression signature we computed the median of all measured chrometric features for all cells in the selected microregions (n = 11). The correlation of the median of the chrometric features and the DZ signature expression was assessed using the Pearson correlation coefficient. Thereby, the ratio of the heterochromatin to euchromatin content was seen to be highly correlated with the DZ expression signature (Pearson r: 0.8843, p-value < 0.0124, non-parametric permutation test, (Supplementary Figure 5G). The heterochromatin content was quantified as the area of the regions of the segmented nuclei where the DAPI intensity was more than one standard deviation above the mean DAPI intensity. Other regions within the nuclei were classified as euchromatin and the sum of their area quantified the euchromatin content of the cells.

Differences of means of the chrometric features in given cell populations were tested for using the Welch t-test. The statistical significance of the correlation measures was identified by applying a non-parametric permutation test using b=100,000 permutations for the correlation analyses of the chromatin state and the DZ gene expression signature respectively b=1,000,000 for the analyses of the chromatin state of B-cells in the context of their distance to the interface. The permutation tests were performed using the python package netneurotools (https://github.com/netneurolab/netneurotools).

Library preparation and sequencing of the Visium spatial transcriptomics

RNA extraction from mouse mesenteric lymph node FFPE blocks was performed using the RNeasy FFPE Kit (Qiagen) according to the manufacturer's instructions. The RNA concentration was determined using a Nanodrop-one spectrophotometer (Thermofisher Scientific), and the average fragment size was estimated using an Agilent Bioanalyzer 2100 (Agilent) with the RNA 6000 Nano kit (Agilent). Samples with a DV200 value greater than 50% were considered suitable for the spatial transcriptomics experiment. The selected samples were sectioned to a thickness of 4 µm, placed on Visium slides and arranged as within the capture area (6.5 × 6.5 mm). Hematoxylin and eosin staining sections were performed and images were acquired using an Aperio CS2 digital slide scanner (Leica Biosystems). Libraries were prepared according to the Visium Spatial Gene Expression for FFPE User Guide, and their quality was assessed using an Agilent Bioanalyzer 2100

with the Agilent High Sensitivity DNA Kit (Agilent). The libraries were sequenced on a NextSeq 2000 Sequencing System (Illumina) using paired-end, dual-indexed sequencing run type, following the sequencing instructions specified in the Visium User Guide.
Bulk-RNA was sequenced following the RNASeq RiboZero - Illumina Stranded Prep protocol on the NextSeq 550 platform. The sequencing parameters were set to 2x75 nt, with a read depth of 50 million reads.

qPCR and RNAseq on ATRi-treated and control DLBCL cell lines
$3x10^6$ HT or SUDHL-5 cells were cultured in 5 mL of RPMI-1640 (Euroclone) plus 10% of Fetal Bovine Serum (FBS), 1% glutamine and 1% of antibiotic-antimycotic solution in the presence of 1 μM ATRi or vehicle (DMSO), as control, for 48 hours at 37°C, 5% CO_2. Cell viability was assessed by MTS assay: $1x10^3$ HT or SUDHL-5 tumor cells were cultured in 200 μL of fresh medium in flat 96-well plates (Corning Inc.) in the presence of 1 μM ATRi or DMSO as control. At 24 hours and 48 hours, 20 μL of 3-(4,5-dimethylthiazol-2-yl)-5- (3-carboxymethoxyphenyl)-2-(4-sulfophenyl)-2H-tetrazolium (MTS) (Promega CellTiter 96 AQueous Nonradioactive Cell Proliferation Assay Kit, Thermo Fisher Scientific) were added to each well. The plates were incubated for 1 hour at 37°C in 5% CO_2, after which absorbance was measured in a spectrophotometer at a wavelength of 490 nm. For qPCR analyses, HT and SUDHL-5 cells were pelleted, washed twice with ice-cold 1X PBS, and RNA extraction was performed for each sample using the RNAeasy Mini kit according to manufacturer instructions. cDNA was prepared for each sample starting from 1μg of RNA using the High-Capacity cDNA Reverse Transcription Kit according to the manufacturer’s instructions. cDNA samples were finally treated with 1μl RNAse H (Promega) for 20 minutes at 37°C and stored at -80 °C. For RNAseq, RNA extraction was performed using the miRNeasy Tissue/Cells Advanced Mini Kit (Qiagen) following the manufacturer’s instructions. For each cell line and treatment conditions, three replicates were profiled. The libraries were constructed using the SureSelect XT HS2 mRNA Library Preparation and SureSelect Poly-A Selection Module kits (Agilent), following the manufacturer's instructions. The libraries were then sequenced using the NextSeq 2000 sequencer with a P3 card, setting the read coverage to 30,000,000 paired-end reads. The processed data were analyzed using the nf-core/rnaseq pipeline, which is available online at https://nf-co.re/rnaseq. The pipeline was run with all default parameters and Ensembl version 109. Gene quantification was performed using the Star-Salmon tool. The GRCh38 genome annotation was considered. The BAM files were converted into read counts using the function summarizeOverlaps from the GenomicAlignments R package(8). log2-counts-per-million normalization was applied using the cpm function of the R limma package.

Quantitative analysis of micronuclei formation in DLBCL cell lines upon ATRi treatment
HT and SUDHL-5 cells treated with ATRi 1μM or DMSO for 48 hours were seeded by centrifugation (5 minutes at 1500 rpm) on 8-well glass-bottom slides (Lab-Tek II) previously coated with poly-D-lysine (for 1 hours at 37°C followed by three washes in sterile 1X PBS). Cells were then washed once with PBS and fixed with 4% formaldehyde (15 minutes at RT), washed 3 times with PBS (10 minutes each) then permeabilized with 0.5% Triton-X-100 in PBS (5 minutes at RT), blocked with 3% BSA in 0.1%triton-X-100 PBS for 1 hour (blocking buffer), incubated with anti-Lamin B1 (1:200 in blocking solution, Synaptic Systems) primary antibody for 2 hours at RT, followed by three PBS washes and then incubated with secondary antibodies (1:400 in blocking solution) for 1 hour in dark at RT followed by three PBS washes. DAPI staining was added in PBS for 5 minutes at RT followed by other 2 washes with PBS. Samples were mounted with Mowiol and stored at 4°C until imaging acquisition. Random fields were acquired from each condition on a Leica Sp8 confocal microscope or, alternatively, on an Olympus Spinning Disk CSU. Z-stacks were acquired for each field of view using 63x oil-immersion objective (NA 1.4) or a 40X oil immersion objective (for the

CSU). The images were then processed using ImageJ and the number of micro-nucleated cells were scored.

Competitive migration assay in microfluidic devices

Before loading the cells, the devices were sterilized under ultraviolet light in a laminar flow hood for 15 minutes and then placed on ice to contrast gel solidification during the filling process. HT and SUDHL-5 tumor cells were resuspended in 2.5 mg/ml Matrigel (Corning). Where indicated, at the cell-matrigel mixture 1µM ATR inhibitor (AZD6738 57693 Selleckchem) or vehicle DMSO were added. Five µL of tumor cell-matrigel mix were loaded in each narrow side chamber, corresponding to $2x10^4$ cells per channel. The devices were incubated at 37°C for 30 minutes to allow gel solidification. Peripheral blood mononuclear cells (PBMC) were isolated from the buffy coat of healthy donors by Lymphosep separation (Aurogene) (9). PBMCs were labelled with PKH26 Red Fluorescence Cell Tracker (Sigma Aldrich), resuspended in RPMI complete medium and loaded (1 x 10^6 cells in 10 µl) into the central chamber of the device. The lateral chambers were filled with medium to keep fluid equilibrium and the devices were placed in a 37°C, 5% CO_2 incubator. Phase contrast, visible and fluorescence photomicrographs were generated at definite time points (0, 24, 48 hours) by using EVOS-FL fluorescence microscope (Life Technologies) provided with built-in imaging software for imaging overlays. Fluorescence analysis of PBMC migration towards and into tumor cell lateral chambers was performed using ImageJ software (National Institutes of Health, Bethesda, MD, USA). Representative regions of interest were analyzed for each device to quantify PBMC migration.

To detect the spatial interaction of $CD3^+$ T cells with DLBCL cells in the microfluidic device, immunofluorescence labeling of T cells infiltrating the tumor cell chamber containing ATRi was performed. At the end of incubation (48 hours), medium was removed from the lateral reservoirs and the central channel to eliminate residual PBMC that did not migrate into the tumor chamber. Each channel was washed with 50 µl PBS and then with 50 µl 1% BSA in PBS. Staining of T cells within the tumor gel chamber containing ATRi was carried out using an anti-human CD3 antibody (Biolegend) diluted 1:200 in PBS for 45 minutes at 4°C, followed by Streptavidin-AlexaFluor488 (Thermo Scientific) (1:100) for 45 minutes at 4°C. The chips were then washed with PBS and fixed with 2% PFA+1% glutaraldehyde solution for 20 minutes. Finally, a DAPI (Invitrogen) staining solution (1:250) was added to label nuclei. Images were taken on a confocal microscope Zeiss LSM 900 (Carl Zeiss) in Airyscan mode. Excitation light was obtained by diode lasers: 405, 488, 561 and 640 nm. Optical thickness varies according to the objective used from 0.50 mm with 20x objective to 0.20 mm with 63x objective. Images have been treated and analysed by the Zen Blue (3.2) software (Carl Zeiss GmbH, Jena Germany) and ImageJ (1.53) software (NIH, USA - http://imagej.nih.gov/ij). LSCM Airyscan mode was also used to measure the distances between HT cells and T cells. Different Z stacks inside the DMSO and ATRi chambers of the microfluidic devices were acquired. We used these stacks to determine the 3D distance between $DAPI^+PKH26^+CD3^+$ and/or $DAPI^+PKH26^+CD3^-$ PBMCs and HT cells ($DAPI^+$ only). Each distance measure is characterized by two points (X, Y, Z coordinates): the centroid of the PBMC and that of the neighboring tumor cells (by using the DAPI fluorescence). We calculated these distances through the three-dimensions Pythagorean theorem[9].

Statistical and bioinformatics analyses

Differential expression analysis: Upregulated/downregulated genes were selected by calculating the limma moderated statistic (10) and applying the Benjamini-Hochberg correction on p-values (BH adjusted p-values < 0.05).

Unsupervised hierarchical clustering: The ward.D2 algorithm was used for unsupervised hierarchical clustering on the z-score normalized gene expression. The heatmaps were used for the clustering

representation. The heatmaps titled with the pathway names (Supplementary Figures 5A,B,H) refer to the gene pathways selected using the Nanostring Panel Pro tool (https://nanostring.com/products/ncounter-assays-panels/panel-selection-tool/).

Pathway enrichment analysis and quantitative gene set enrichment analysis (GSEA): The Reactome Pathway library was considered for the pathway enrichment analysis applied to differentially expressed genes (Figures 1H-I, 4N-O). The enrichment p-values were calculated through the ReactomePA R package(11) and adjusted for multiple comparisons (BH adjusted p-values < 0.05). For GSEA analysis, we run the GSEA software(12) (v 4.2.3) considering the DZ/LZ spatial signature, Nanostring Panel Pro pathways, and the Reactome pathways as input gene sets. The GSEA "iceberg" enrichment plots were used for the enrichment representation (Figures 4M, 5D-E, Supplementary Figure 4E). The plots were generated in GraphPad Prism using the normalized enrichment score (NES) obtained from the GSEA output. The GSEA p-values were calculated using the gene set method with 5000 permutations.

DZ/LZ Single-cell RNAseq analysis: The single-cell analysis was performed on Holmes et al. data from GEO (GSE139891). DZ cells (GSM4148372) and LZ cells (GSM4148374) were considered. Seurat (4.3.0)(13) was used for the analysis of the single-cell datasets. We filtered low-quality cells that have unique feature counts over 5000 or less than 200 and have >5% mitochondrial counts. To compare the predictive power of the spatial DZ/LZ signature and the differentially expressed genes (DEGs) identified through the WTA DSP, we combined Holm's normalized expression of DZ and LZ genes. Based on this score we classified germinal center B cells into DZ and LZ categories (a detailed description of the score is provided later in this paragraph).

DLBCL gene expression datasets: We considered 8 DLBCL cohorts, Visco et al. (GSE31312)(14), Lenz et al. (GSE10846)(15), Dubois et al. (GSE87371)(16), Sha et al. (GSE117556)(17), Barrans et al. (GSE32918)(18), Reddy et al.(19), Schmitz et al.(20), and Chapuy et al. (GSE98588)(21). The Visco, Lenz, Dubois, Chapuy, and Sha datasets have been downloaded from GEO using the GEOquery R package(22). All the datasets have been independently analyzed. The datasets of Sha, Barrans, Schmitz, and Reddy have been analyzed maintaining the normalization proposed by the authors. Regarding the Affymetrix datasets of Visco, Lenz, Dubois, and Chapuy; the expression matrices have been obtained from the CEL files available on GEO and then have been normalized using the gcrma R package(23). The few outlier DLBCL samples based on PCA analyses were excluded. After verifying the affinity of Sha et al. and Barrans et al. datasets in terms of normalized gene expression distribution, Sha at al. and Barrans et al. datasets were harmonized using the removeBatchEffect function of the limma package. We have verified the absence of batch effect in the Illumina harmonized dataset running a PCA analysis.

Immune and stromal deconvolution analysis*:* The SpatialDecon algorithm(24) was applied to the DSP dataset to estimate the cell fractions considering the safeTME matrix as reference of the SpatialDecon algorithm. The xCell algorithm(25) was used for immune and stromal deconvolution estimation of cytotype scores on bulk RNA-seq samples. The DZ enrichment score in Figure 3A indicates the association between DZ gene expression and xCell cytotype scores. The DZ enrichment score is calculated as the log2-FC of the xCell score from the comparison between the low DZ expression and high DZ expression DLBCL groups. The low DZ expression and high DZ expression groups were obtained by dividing the cases of each DLBCL dataset into 3 groups based on DZ gene expression tertiles. The Mann-Whitney-Wilcoxon test was used to statistically compare low DZ expression and high DZ expression groups. The p-values were adjusted for multiple comparisons (BH adj. p-values < 0.05).

LZ-like/DZ-like sample classification: The LZ-like, DZ-like, and intermediate groups were obtained by ranking the samples based on the following score:

$$score = \sum_{i=1}^{n} x_i \cdot I_i$$

with,

$$I_i = \begin{cases} -1 & \text{if } logFC_i < 0 \\ +1 & \text{if } logFC_i > 0 \end{cases}$$

where *FCi* is the fold-change of the gene-*i* from the DZ/LZ spatial signature, *xi* is the expression of gene-*i* in the dataset under exam, and *n* is the number of genes of the DZ/LZ spatial signature. The tertile approach was applied to this score to obtain the three-group separation: the LZ-like patients have a high expression of LZ genes and a low expression of DZ genes, the DZ-like patients have a high expression of DZ genes and a low expression of LZ genes, and the intermediate patients have an intermediate expression of both DZ and LZ genes. The PDX sample classification into DZ-like and LZ-like groups was further investigated through a GSVA analysis(26). The GSVA results were consistent with our classification. Moreover, we also applied the tertile approach on the total expression of the DZ spatial signature to obtain the high DZ expression and low DZ groups compared in Supplementary Figure 3B-E.

Survival analysis on DLBCL datasets: The overall survival (OS) and the progression-free survival (PFS) were compared among the DLBCL groups. Kaplan-Meier method was used to estimate the survival functions among groups. The log-rank test was used to test the differences in the OS and PFS among groups. The cox-pzh test was applied to test the proportional hazard assumption. The survival and survminer R packages were used for the data analysis.

The cytotoxic CD8 T cells signature [CD8A, CD8B, PRF1, GZMA, GZMB] and T-cells signature [CD3D, CD3E, UBASH3A, CD2, TRBC2] were supervised selected. To assess the specificity of those signatures, we compared their overall expression levels between T-cell and non-T-cell populations in the TABULA SAPIENS Immune dataset(27). We found that those signatures clearly discriminate T-cell populations from non-T-cell populations (data not shown).

Visium spatial transcriptomics analysis: Visium ST (10x Genomics) samples were aligned using Space Ranger (10x Genomics) and mapped to the reference genome. The gene expression count matrices were analyzed through Seurat(28) (v.4.3.0). We separately normalized the two samples using the SCTransform. Clustering was performed using the FindClusters function. Cluster markers were identified using the two-sided Wilcoxon rank-sum test of the FindAllMarkers function. P values were adjusted by Benjamini-Hochbergh correction (BH adj. p-value < 0.05, log-FC > 0.25).

Software

Statistical analyses were performed using R software (v 4.3.0) (http://www.R-project.org). GSEA (v 4.2.3) was used for calculating NESs and enrichment p-values. The chromatic features analysis and the machine learning approach were implemented using the Python software (v. 3.12.2). The figures were produced using the R software, Python software, and Prism GraphPad (v 10.0.1).

SUPPLEMENTARY FIGURES

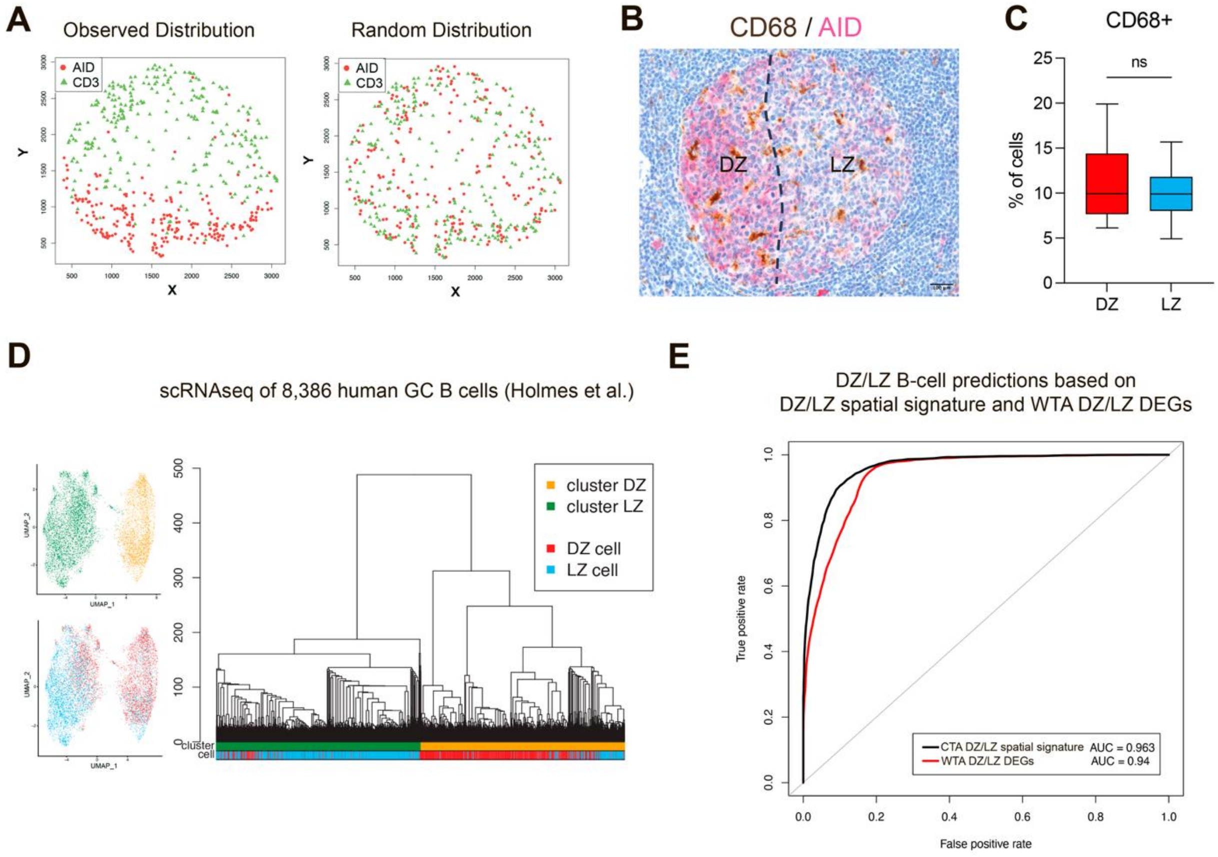

Supplementary Figure 1

A, Example of observed and randomized spatial distribution.

B-C, Representative microphotographs, quantitative analyses of double-marker IHC for CD68$^+$ (brown signal) and AID$^+$ (pink signal) cells (*n* GCs = 20). Original magnification, x200. Scale bars, 100 µm. Statistical analysis: two-tailed unpaired Mann-Whitney test (**C**). Statistical analysis was assessed using a two-tailed unpaired Mann-Whitney test. Values are shown as mean ± standard error.

D, UMAP projection of GC B cells from Holmes et al. (GSE139891), clustered by expression of the spatial DZ/LZ signature. DZ and LZ signature–based clusters (orange and green) overlap with annotated DZ (red) and LZ (blue) populations.

E, Comparison of the predictive power of the spatial DZ/LZ signature and the WTA-derived DZ/LZ DEGs in classifying germinal center B cells into DZ and LZ types. The spatial signature demonstrates higher accuracy, confirming its strong discriminatory capacity.

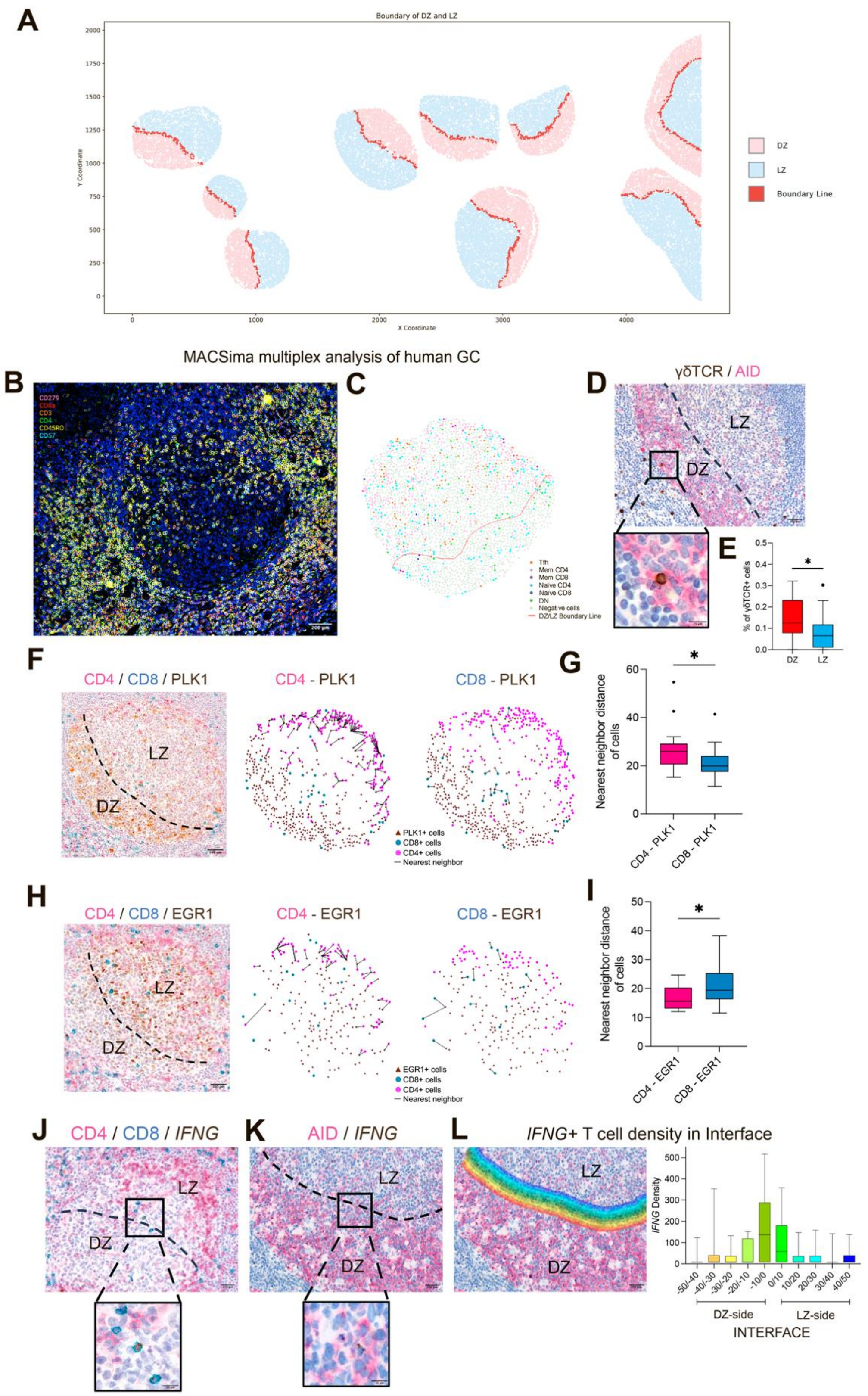

Supplementary Figure 2

A, DZ and LZ spatial signature and boundary line mapping on nine GCs analysed by CosMx WTX. **B-C,** Hyperplexed MACSima image (**B**) and spatial plot (**C**) of a representative germinal center. A subset of markers including CD279, CD8α, CD3, CD4, CD45RO, CD57, was used to identify and spatially resolve distinct T cell populations. Original magnification, x100. Scale bars, 200 μm.

D-E, Representative microphotograph (**D**) and quantitative analyses (**E**) of double-marker IHC for γδTCR$^+$ (brown signal) and AID$^+$ (pink signal) cells showing different spatial enrichment and expression in DZ and LZ (*n* GCs = 20). Original magnification, x100 and x630 (insets). Scale bars, 200 μm and 25 μm.

F, Representative microphotographs of nearest neighbor average distance showing the proximity of PLK1$^+$ (brown triangle) to CD4$^+$ (pink circle) and CD8$^+$ cells (blue circle). Original magnification, x100 and x200. Scale bars, 200 μm and 100 μm.

G, Quantification of nearest neighbor distances in (**F**).

H, Representative microphotographs of nearest neighbor average distance showing the proximity of EGR1$^+$ (brown triangle) to CD4$^+$ (pink circle) and CD8$^+$ cells (blue circle). Original magnification, x100 and x200. Scale bars, 200 μm and 100 μm.

I, Quantification of nearest neighbor distances in (**H**).

J, Representative microphotographs of combined mRNA *in situ* hybridization of *IFNG* (brown signal) and double-marker immunohistochemistry of CD4 (pink signal) and CD8 (green signal). Original magnification, x200 and x630 (insets). Scale bars, 100 μm and 25 μm.

K-L, *In situ* detection for *IFNG* mRNA (brown signal) and IHC for AID (pink signal) representative images (**K**), DZ/LZ infiltration analysis representation and quantitative analyses of the average density of *IFNG* cells infiltrating the inside and outside of the interface (**L**). (*n* GCs = 10). Original magnification, x200 and x630 (insets). Scale bars, 100 μm and 25 μm.

Statistical analysis: two-tailed unpaired Mann-Whitney test. Mean ± standard error shown; *, $P < 0.05$.

A

1078 DLBCL Cases (GSE117556; GSE32918)	Follow Up (months)	0	12	24	36	48	60
LZ-Like	Risk Population	359	321	229	130	61	33
	Survival	1	0.92	0.88	0.84	0.83	0.78
	CI	-	0.9-0.95	0.84-0.91	0.8-0.89	0.78-0.88	0.72-0.85
Intermediate	Risk Population	360	299	207	111	55	20
	Survival	1	0.88	0.82	0.8	0.77	0.63
	CI	-	0.84-0.91	0.78-0.86	0.76-0.85	0.71-0.83	0.53-0.77
DZ-Like	Risk Population	359	282	187	106	56	19
	Survival	1	0.81	0.72	0.69	0.68	0.65
	CI	-	0.77-0.85	0.67-0.77	0.64-0.74	0.63-0.73	0.59-0.72

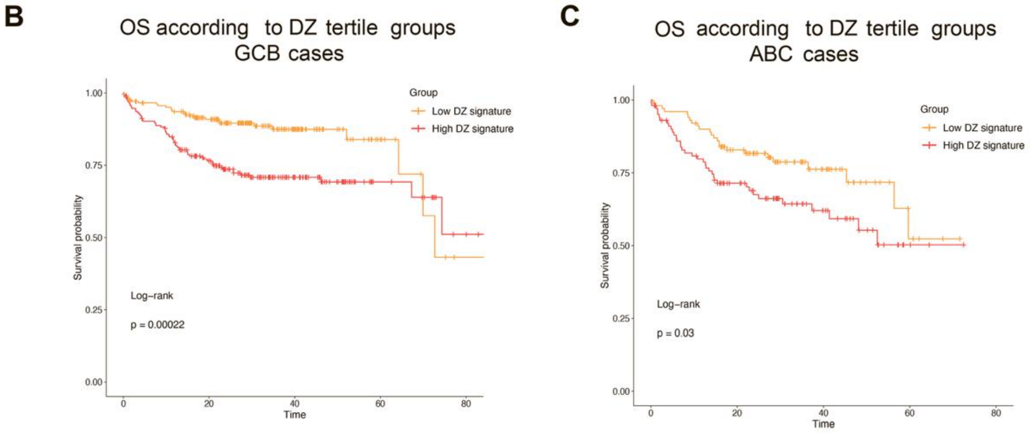

D

GCB Cases	Follow Up (months)	0	12	24	36	48	60
Low DZ Signature	Risk Population	206	184	129	72	29	11
	Survival	1	0.94	0.9	0.87	0.87	0.84
	CI	-	0.9-0.97	0.85-0.94	0.82-0.93	0.82-0.93	0.76-0.93
High DZ Signature	Risk Population	206	166	115	73	38	14
	Survival	1	0.83	0.74	0.71	0.69	0.69
	CI	-	0.78-0.88	0.68-0.8	0.65-0.78	0.62-0.77	0.62-0.77

E

ABC Cases	Follow Up (months)	0	12	24	36	48	60
Low DZ Signature	Risk Population	102	89	62	32	13	5
	Survival	1	0.89	0.82	0.76	0.72	0.52
	CI	-	0.83-0.95	0.74-0.9	0.67-0.86	0.6-0.85	0.53-0.84
High DZ Signature	Risk Population	102	77	50	29	15	3
	Survival	1	0.79	0.66	0.64	0.55	0.5
	CI	-	0.71-0.87	0.57-0.77	0.55-0.75	0.44-0.7	0.37-0.68

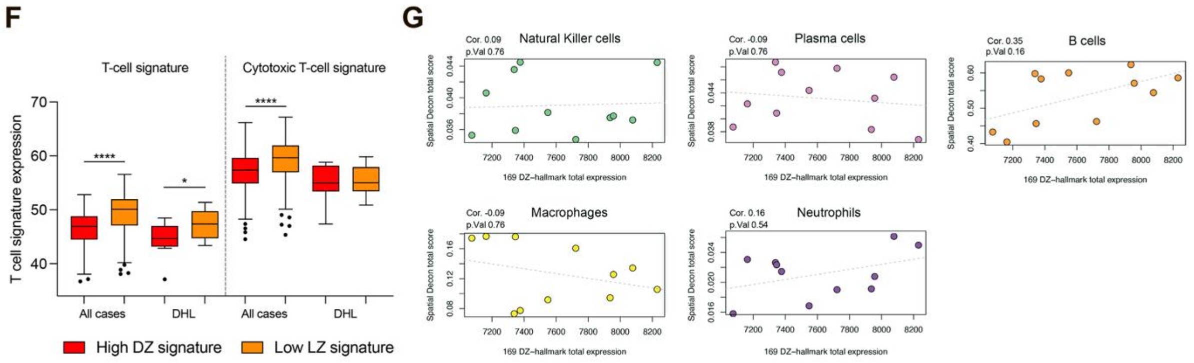

Supplementary Figure 3

A, Overall survival values over DZ-like, LZ-like, and intermediate patients from the harmonized dataset (1078 cases).

B-E, Overall survival curves and values in 618 GCB (**B, D)** / 305 ABC (**C,E**) DLBCL harmonized cases for high DZ expression and low DZ expression patient groups obtained from tertile separation of the DZ spatial signature expression.

F, Expression of T-cell signatures over DLBCL patients and the double-hit lymphoma cases (DHL). Wilcoxon p-values have been calculated to compare the T-cell gene expression between DZ high expression and DZ low expression patients. Statistical analysis: two-tailed unpaired Mann-Whitney test. Mean ± standard error shown.

G, Association between DZ spatial signature expression and SpatialDecon cytotype scores across 11 IG ROIs (Kendall's correlation, p-value < 0.05).

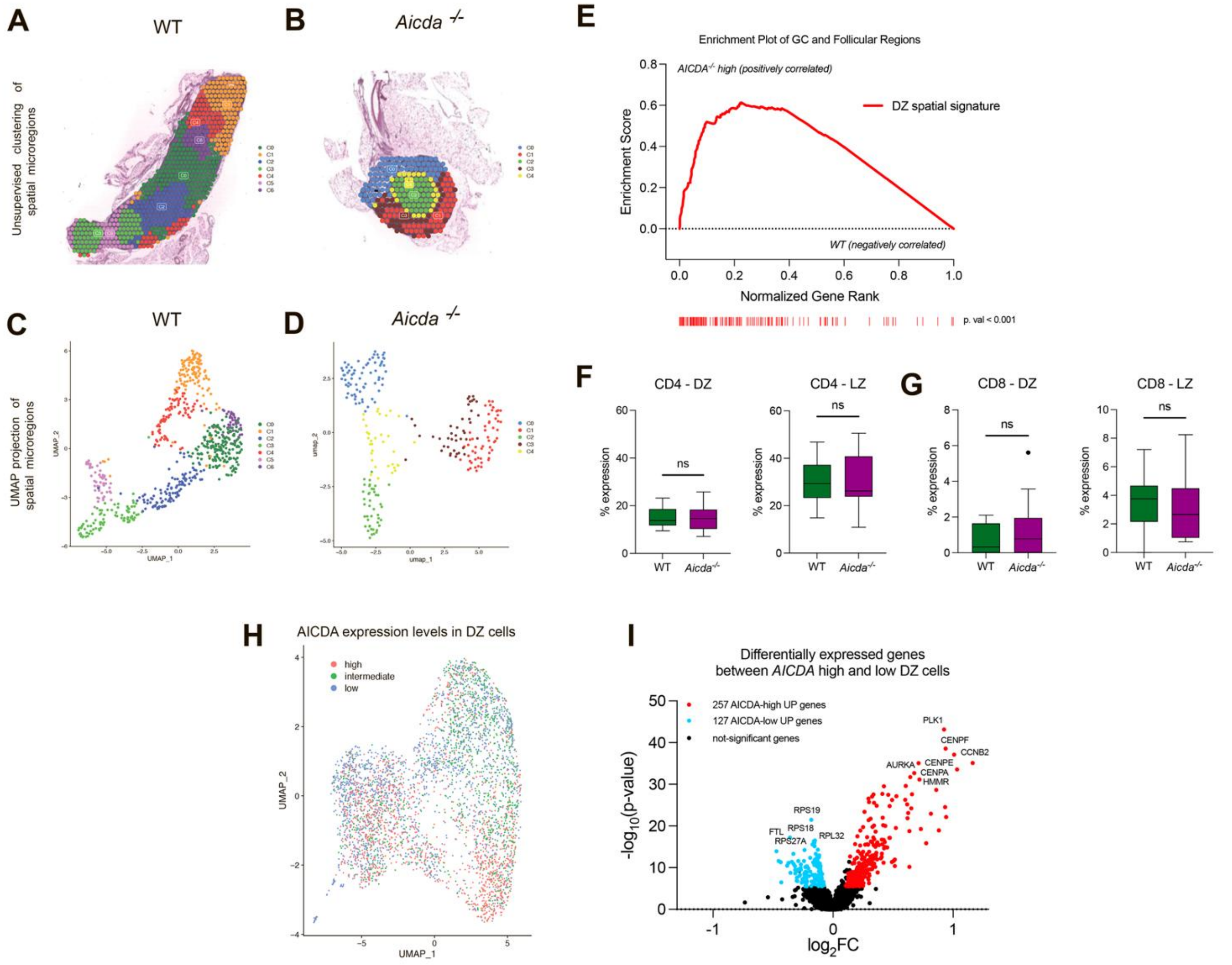

Supplementary Figure 4

A-B, Unsupervised clustering of spatial microregions in WT (**A**) and *Aicda* $^{-/-}$ (**B**) mLNs.

C-D, UMAP projection of the spatial microregions in WT (**C**) and *Aicda* $^{-/-}$ (**D**) mLNs. Colors reflect the unsupervised cluster classification.

E, GSEA enrichment analysis on follicular-GC microregions. The spatial DZ spatial signatures significantly enriches follicular-GC regions of the *Aicda* $^{-/-}$ sample (p-value < 0.001).

F-G, Quantitative analyses of the percentage of $CD4^+$ (**G**) or $CD8^+$ (**H**) T cells in DZ and LZ GCs. (*n* = 10 WT GCs; *n* = 10 *Aicda* $^{-/-}$ GCs). Statistical analysis: two-tailed unpaired Mann-Whitney test. Mean ± standard error shown.

H, UMAP projection of 4.082 cells from the Holmes et al. dataset. The cells are classified as low, intermediate, and high AICDA gene expression. While low indicates the absence of expression, and high indicates an expression greater than the 2nd tertile.

I, Volcano plot showing differentially expressed genes between AICDA-high and AICDA-low cells from the Holms et al. single-cell dataset (Wilcoxon Rank Sum test adj. p-value < 0.05, abs-logFC > 0.25).

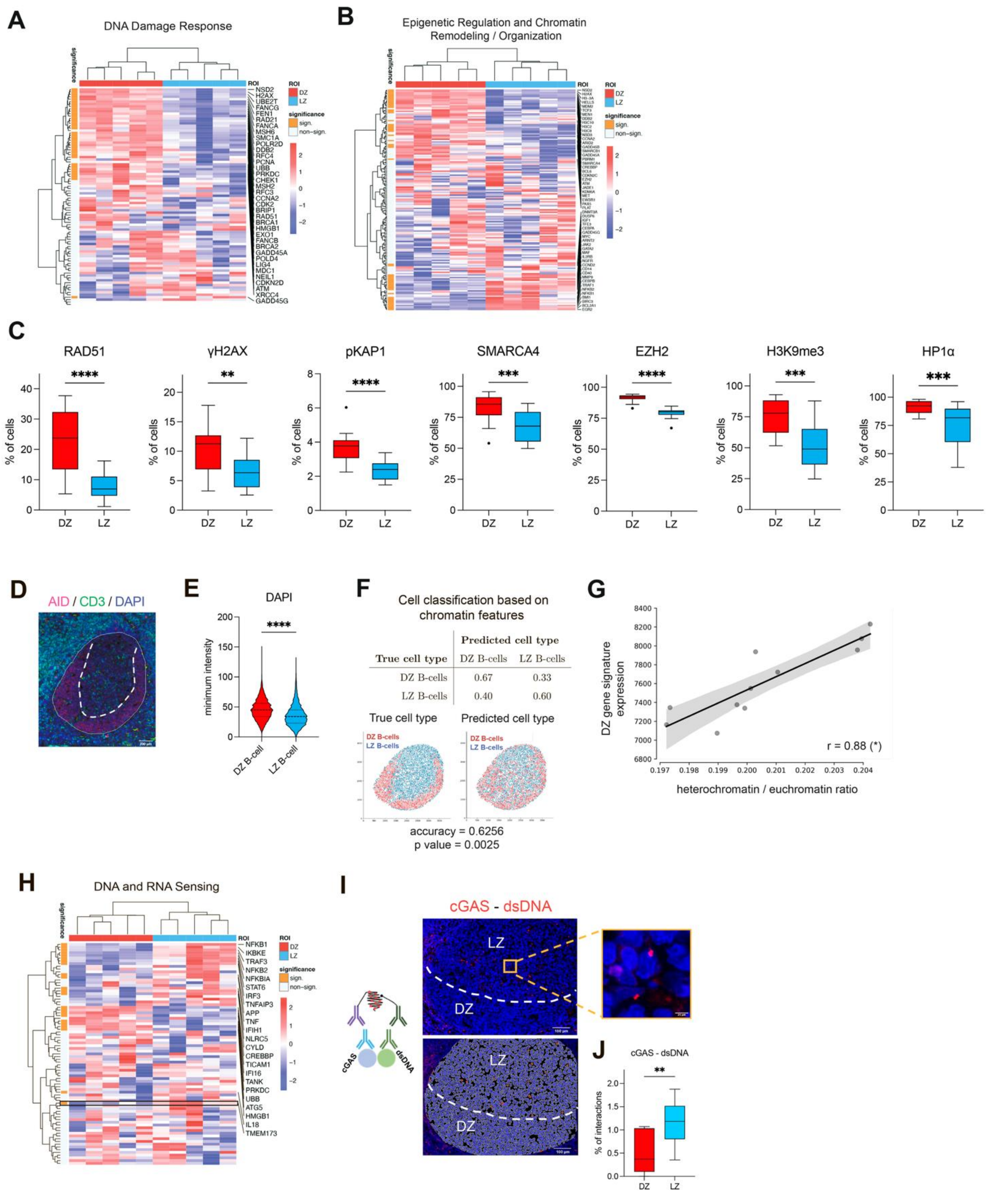

Supplementary Figure 5

A, Expression of DNA Damage Response genes in DZ and LZ ROIs. The left bar indicates the significant DEGs between DZ and LZ ROIs.

B, Expression of Epigenetic Regulation and Chromatin Remodeling/Organization genes in DZ and LZ ROIs. The left bar indicates the significant DEGs between DZ and LZ ROIs.

C, Quantitative analyses of RAD51, γH2AX, pKAP1, SMARCA4, EZH2, H3K9me3 or HP1α expression to assess the different enrichment between DZ and LZ. (*n* GCs = 20).

D, Representative microphotographs of a GC showing the AID (red signal) and CD3 (green signal) staining. Original magnification x100. Scale bar, 200 µm.
E, Violin plot showing the distribution of the "minimum DNA intensity" among the DZ/LZ B-cell populations (Welch's t-test, p-value < 1e-124).
F, Average of the row-normalized confusion matrices of the RFC trained to distinguish between LZ and DZ B-cells. The average is obtained by evaluating the RFC in a 10-fold stratified cross-validation setup for a balanced random subsample of DZ and LZ B-cells (*n* = 9,197). The prediction accuracy (Acc = 0.635) is significantly higher than the No Information Rate (NIR = 0.5, p-value 0.0025, one-sided Wilcoxon signed-rank test) (top). Visualization of the prediction performance of the RFC for a GC sample. The true cell-type labels are shown on the left. Cell type labels predicted by an RFC when holding out the respective nuclei during training of the RFC are shown on the right.
G, Scatterplot shows the measured DZ gene signature expression of the ROIs (*n* = 11) plotted against the median heterochromatin-to-euchromatin (HC/EC) ratio of the nuclei in those regions. The black line shows the fit of a linear regression model which visualises the significant correlation of the two quantities (Pearson r=0.8843, p-value = 0.0180, permutation test). A 95% confidence interval computed using 1,000 bootstrap samples for the regression line is shown as the shaded region in grey (bottom).
H, Expression of "DNA and RNA sensing" genes in DZ and LZ ROIs. The left bar indicates the significant DEGs between DZ and LZ ROIs.
I-J, Representative microphotographs and segmentation image (**I**) and quantitative analyses (**J**) showing cGAS/dsDNA interactions (red signal) detected by fluorescent *in situ* proximity ligation assay (*n* GCs = 10) and showing scattered elements in the LZ regions. Original magnification, x200 and x630 (insets). Scale bars, 100 µm and 10 µm. Statistical analysis: two-tailed unpaired Mann-Whitney test. Mean ± standard error shown; **, P < 0.01; ***, P < 0.001; ****, P < 0.0001.

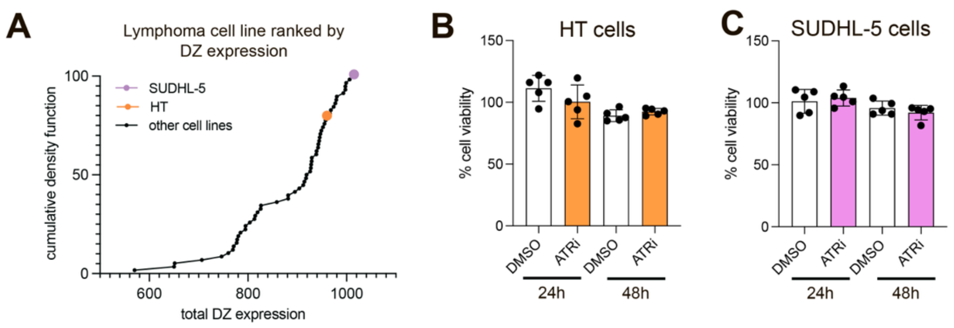

Supplementary Figure 6

A, Lymphoma cell line ranking based on the total expression of DZ spatial signature genes. The SUDHL5 and HT cell lines are in the top-ranking positions.

B-C, MTT assay show that the viability of HT (**B**) and SUDHL-5 (**C**) DZ-like DLBCL cells was not selected along 48h culture with ATRi treatment (1 micromolar). Statistical analysis: two-tailed unpaired Mann-Whitney test. Mean ± standard error shown.

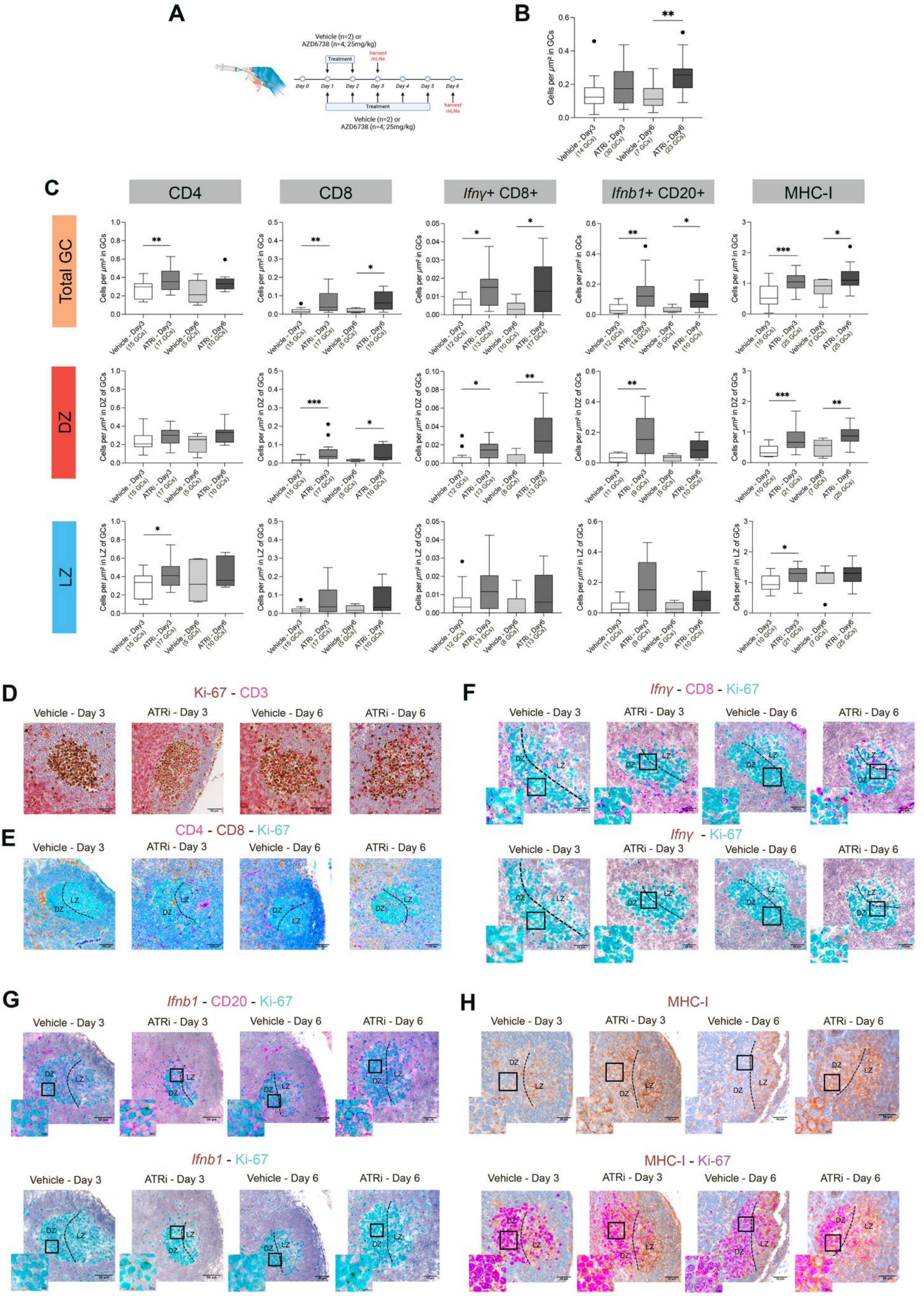
A
Vehicle (n=2) or
AZD6738 (n=4; 25mg/kg)
Treatment
harvest
mLNs
Day 0
Day 1
Day 2
Day 3
Day 4
Day 5
Day 6
Treatment
Vehicle (n=2) or
AZD6738 (n=4; 25mg/kg)
B
Cells per μm² in GCs
Vehicle - Day3 (14 GCs)
ATRi - Day3 (30 GCs)
Vehicle - Day6 (7 GCs)
ATRi - Day6 (23 GCs)
C
CD4
CD8
Ifnγ+ CD8+
Ifnb1+ CD20+
MHC-I
Total GC
DZ
LZ
Cells per μm² in GCs
Cells per μm² in DZ of GCs
Cells per μm² in LZ of GCs
D
Ki-67 - CD3
Vehicle - Day 3
ATRi - Day 3
Vehicle - Day 6
ATRi - Day 6
E
CD4 - CD8 - Ki-67
F
Ifnγ - CD8 - Ki-67
Ifnγ - Ki-67
G
Ifnb1 - CD20 - Ki-67
Ifnb1 - Ki-67
H
MHC-I
MHC-I - Ki-67
DZ
LZ

Supplementary Figure 7

A, Schematic of ATRi or vehicle control treatment for 2 or 5 consecutive days in BALB/c mice.

B, Quantitative analyses of the number of $CD3^+$ T cells per GCs area, expressed in μm^2, in control and ATRi-treated mice.

C, Quantitative analysis of the number of $CD4^+$, $CD8^+$, *Ifnγ*$^+$$CD8^+$, *Ifnb1*$^+$$CD20^+$, and MHC-$I^+$ cells per GC area (expressed in μm^2) in vehicle control and ATRi-treated mice at day 3 and day 6.

Statistical analysis: two-tailed unpaired Mann-Whitney test. Mean ± standard error shown; *, $P < 0.05$; **, $P < 0.01$; ***, $P < 0.001$.

D, Double-marker IHC of Ki-67^+ (brown signal) and $CD3^+$ (pink signal) cells, in vehicle control and ATRi-treated mice at day 3 and day 6. Original magnifications x400. Scale bar, 50 µm.

E, Triple immunohistochemical staining for $CD4^+$ (pink signal), $CD8^+$ (brown signal) and Ki-67^+ cells (cyan signal) in vehicle control and ATRi-treated mice at day 3 and day 6. Original magnifications x200. Scale bar, 100 µm.

F, Representative microphotographs of combined mRNA *in situ* hybridization of *Ifnγ* (brown signal) and double-marker immunohistochemistry of CD8 (pink signal) and Ki-67 (cyan signal) or *Ifnγ* (brown signal) and Ki-67 (cyan signal) in vehicle control and ATRi-treated mice at day 3 and day 6. Original magnification, x400 and x630 (insets). Scale bars, 50 µm and 25 µm.

G, Representative microphotographs of combined mRNA *in situ* hybridization of *Ifnb1* (brown signal) and double-marker immunohistochemistry of CD20 (pink signal) and Ki-67 (cyan signal) or *Ifnb1* (brown signal) and Ki-67 (cyan signal) cells in vehicle control and ATRi-treated mice at day 3 and day 6. Original magnification, x400 and x630 (insets). Scale bars, 50 µm and 25 µm.

H, Representative images of IHC for MHC-I (brown signal) or double-marker IHC of MHC-I (brown signal) and Ki-67 (pink signal) in vehicle control and ATRi-treated mice at day 3 and day 6. Original magnification, x400 and x630 (insets). Scale bars, 50 µm and 25 µm.

REFERENCES

1. Bertolazzi G, et al. Resampling approaches for the quantitative analysis of spatially distributed cells. *Data Intell*. 2024;6(1):104–119.

2. Schmidt U, et al. Cell detection with star-convex polygons. *arXiv [csCV]*. 2018. http://arxiv.org/abs/1806.03535.

3. Venkatachalapathy S, Jokhun DS, Shivashankar GV. Multivariate analysis reveals activation-primed fibroblast geometric states in engineered 3D tumor microenvironments. *Mol Biol Cell*. 2020;31(8):803–812.

4. Venkatachalapathy S, et al. Single cell imaging-based chromatin biomarkers for tumor progression. *Sci Rep*. 2021;11(1). https://doi.org/10.1038/s41598-021-02441-6.

5. Breiman L. Random Forests. *Mach Learn*. 2001;45(1):5–32.

6. Brodersen KH, et al. The balanced accuracy and its posterior distribution. *2010 20th International Conference on Pattern Recognition*. IEEE; 2010.

7. Pedregosa F, et al. Scikit-learn: Machine Learning in Python. *arXiv [csLG]*. 2012. http://arxiv.org/abs/1201.0490.

8. Lawrence M, et al. Software for computing and annotating genomic ranges. *PLoS Comput Biol*. 2013;9(8):e1003118.

9. Lucarini V, et al. Combining type I interferons and 5-Aza-2′-deoxycitidine to improve anti-tumor response against melanoma. *J Invest Dermatol*. 2017;137(1):159–169.

10. Ritchie ME, et al. limma powers differential expression analyses for RNA-sequencing and microarray studies. *Nucleic Acids Res*. 2015;43(7):e47–e47.

11. Yu G, He Q-Y. ReactomePA: an R/Bioconductor package for reactome pathway analysis and visualization. *Mol Biosyst*. 2016;12(2):477–479.

12. Subramanian A, et al. Gene set enrichment analysis: A knowledge-based approach for interpreting genome-wide expression profiles. *Proc Natl Acad Sci U S A*. 2005;102(43):15545–15550.

13. Hao Y, et al. Integrated analysis of multimodal single-cell data. *Cell*. 2021;184(13):3573-3587.e29.

14. Visco C, et al. Comprehensive gene expression profiling and immunohistochemical studies support application of immunophenotypic algorithm for molecular subtype classification in diffuse large B-cell lymphoma: a report from the International DLBCL Rituximab-CHOP Consortium Program Study. *Leukemia*. 2012;26(9):2103–2113.

15. Lenz G, et al. Stromal gene signatures in large-B-cell lymphomas. *N Engl J Med*. 2008;359(22):2313–2323.

16. Dubois S, et al. Biological and clinical relevance of associated genomic alterations in MYD88 L265P and non-L265P-mutated diffuse large B-cell lymphoma: Analysis of 361 cases. *Clin Cancer Res*. 2017;23(9):2232–2244.

17. Sha C, et al. Molecular high-grade B-cell lymphoma: Defining a poor-risk group that requires different approaches to therapy. *J Clin Oncol*. 2019;37(3):202–212.

18. Barrans SL, et al. Whole genome expression profiling based on paraffin embedded tissue can be used to classify diffuse large B-cell lymphoma and predict clinical outcome. *Br J Haematol*. 2012;159(4):441–453.

19. Reddy A, et al. Genetic and functional drivers of diffuse large B cell lymphoma. *Cell*. 2017;171(2):481-494.e15.

20. Schmitz R, et al. Genetics and pathogenesis of diffuse large B-cell lymphoma. *N Engl J Med*. 2018;378(15):1396–1407.

21. Chapuy B. Molecular subtypes of diffuse large B cell lymphoma are associated with distinct pathogenic mechanisms and outcomes. *Nat Med*. 2018;24(5):1292-E98588.

22. Davis S, Meltzer PS. GEOquery: a bridge between the Gene Expression Omnibus (GEO) and BioConductor. *Bioinformatics*. 2007;23(14):1846–1847.

23. Wu J, Riwcfjmj G. *gcrma: Background Adjustment Using Sequence Information*. 2023.

24. Griswold M, Danaher P. *SpatialDecon: Deconvolution of mixed cells from spatial and/or bulk gene expression data*. 2022.

25. Aran D, Hu Z, Butte AJ. xCell: digitally portraying the tissue cellular heterogeneity landscape. *Genome Biol*. 2017;18(1). https://doi.org/10.1186/s13059-017-1349-1.

26. Hänzelmann S, Castelo R, Guinney J. GSVA: gene set variation analysis for microarray and RNA-seq data. *BMC Bioinformatics*. 2013;14(1):7.

27. Tabula Sapiens Consortium*, et al. The Tabula Sapiens: A multiple-organ, single-cell transcriptomic atlas of humans. *Science*. 2022;376(6594):eabl4896.

28. Satija R, et al. Spatial reconstruction of single-cell gene expression data. *Nat Biotechnol*. 2015;33(5):495–502.
